# Accessing a synthetic Fe^III^Mn^IV^ core to model biological heterobimetallic active sites†

**DOI:** 10.1039/d3sc04900k

**Published:** 2023-12-27

**Authors:** Justin L. Lee, Saborni Biswas, Joseph W. Ziller, Emile L. Bominaar, Michael P. Hendrich, A. S. Borovik

**Affiliations:** a Department of Chemistry, University of California–Irvine Irvine CA 92697 USA aborovik@uci.edu; b Department of Chemistry, Carnegie Mellon University Pittsburgh PA 15213 USA

## Abstract

Metalloproteins with dinuclear cores are known to bind and activate dioxygen, with a subclass of these proteins having active sites containing FeMn cofactors and activities ranging from long-range proton-coupled electron transfer (PCET) to post-translational peptide modification. While mechanistic studies propose that these metallocofactors access Fe^III^Mn^IV^ intermediates, there is a dearth of related synthetic analogs. Herein, the first well-characterized synthetic Fe^III^–(μ-O)–Mn^IV^ complex is reported; this complex shows similar spectroscopic features as the catalytically competent Fe^III^Mn^IV^ intermediate X found in Class Ic ribonucleotide reductase and demonstrates PCET function towards phenolic substrates. This complex is prepared from the oxidation of the isolable Fe^III^–(μ-O)–Mn^III^ species, whose stepwise assembly is facilitated by a tripodal ligand containing phosphinic amido groups. Structural and spectroscopic studies found proton movement involving the Fe^III^Mn^III^ core, whereby the initial bridging hydroxido ligand is converted to an oxido ligand with concomitant protonation of one phosphinic amido group. This series of FeMn complexes allowed us to address factors that may dictate the preference of an active site for a heterobimetallic cofactor over one that is homobimetallic: comparisons of the redox properties of our FeMn complexes with those of the di-Fe analogs suggested that the relative thermodynamic ease of accessing an Fe^III^Mn^IV^ core can play an important role in determining the metal ion composition when the key catalytic steps do not require an overly potent oxidant. Moreover, these complexes allowed us to demonstrate the effect of the hyperfine interaction from non-Fe nuclei on ^57^Fe Mössbauer spectra which is relevant to MnFe intermediates in proteins.

## Abstract 2

Characterization of a synthetic mimic for an enzymatic Fe^III^Mn^IV^ intermediate and its reactivity with phenolic substrates.

## Graphical abstract

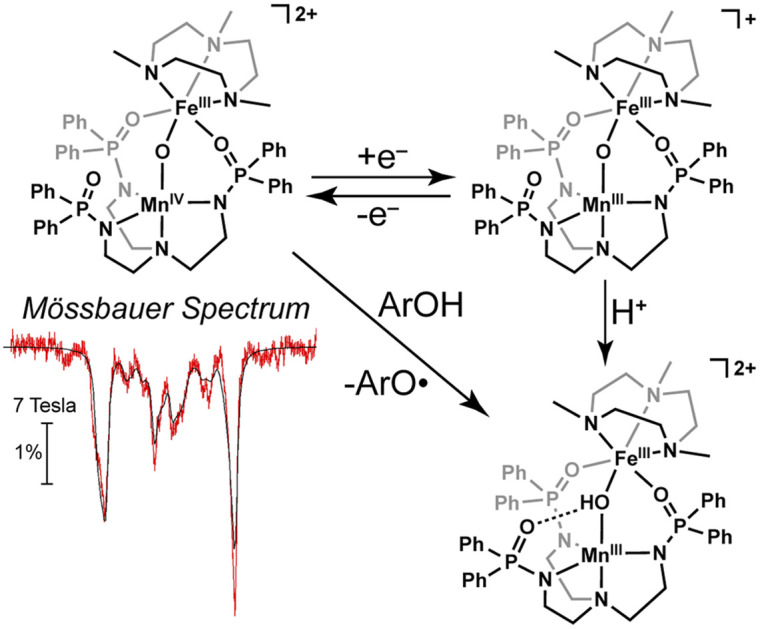

## Introduction

Many dioxygen-binding and/or activating non-heme metalloproteins contain a dinuclear active site, with the majority of them utilizing a diiron core, including the hydroxylase component of soluble methane monooxygenase (sMMO) and the R2 subunit of Class Ia ribonucleotide reductase (RNR).^1–5^ There is an increasing number of reports on dinuclear enzymes that employ a heterobimetallic FeMn active site to reduce O_2_, such as Class Ic RNR^6–14^ and R2-like ligand-binding oxidase (R2lox).^15–19^ The reproducible coordination of one Fe ion and one Mn ion in designated sites is unusual: Fe is more abundant than Mn under physiological conditions,^20,21^ and the Irving–Williams series supports stronger binding of Fe^II^ over Mn^II^, so an Fe^II^Fe^II^ active site is thermodynamically more likely to form than an Fe^II^Mn^II^ one.^22^ It has been proposed that the tertiary structures of proteins enforce the site-specific binding of these metal ions,^17^ but the thermodynamic requirements for enzymatic functions (*e.g.*, the relative accessibility of various oxidation levels of Fe *vs.* Mn) may also serve as a driving force for the metal selectivity, and these factors are underexplored. Synthetic model compounds are useful to address these fundamental considerations, but the preparation of heterobimetallic complexes is synthetically challenging: few examples of FeMn systems have been reported relative to their homobimetallic counterparts,^23–30^ and their oxidative chemistry has rarely been investigated.^31–35^ In this work, we describe a series of FeMn complexes in the same ligand architecture and explore their proton transfer, electron transfer, and proton-coupled electron transfer (PCET) properties. We examine a high-valent complex that contains an Fe^III^–(μ-O)–Mn^IV^ core that is relevant to key intermediates found for Class Ic RNR and R2lox.

**Scheme 1:**
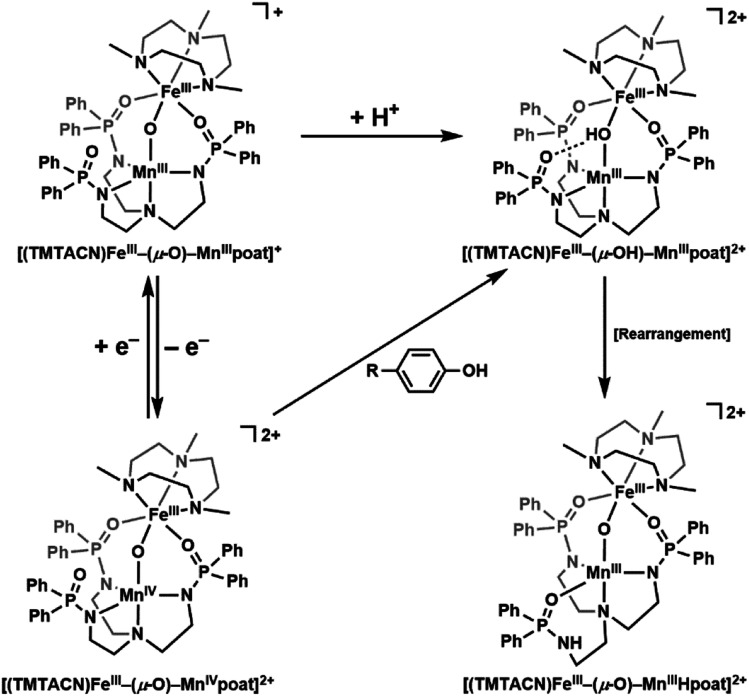
Individual or coupled proton and electron transfers in the FeMn complexes described in this work.

We have previously reported the usage of the multifunctional ligand [poat]^3−^ (*N*,*N*′,*N*′′-[nitrilotris-(ethane-2,1-diyl)]tris(*P*,*P*-diphenylphosphinic amido))^36–40^ in which the *C*_3_ trianionic framework can support a metal center up to the 4+ oxidation level in high spin states. Our studies have shown that the phosphinic amido groups can form intramolecular hydrogen bonds (H-bonds),^38^ act as sites for proton storage, and be part of an auxiliary metal ion binding site, allowing us to construct discrete unsymmetrical bimetallic complexes. We have systematically varied the identity of the auxiliary metal center in a series of M/Fe dinuclear compounds (M = first row transition metals, group II alkali earth metals) to probe their effects on the electronic structures of Fe^IV^=O units^36^ as well as magnetic and redox phenomena,^37^ and prepared a series of di-Fe complexes with the general formulation [(TMTACN)Fe^*m*^–(μ-O(H))–Fe^*n*^(H)poat]^*z*+^ (TMTACN = 1,4,7-trimethyl-1,4,7-triazacyclononane; *m* = II, III; *n* = II, III, IV; *z* = 0, 1, 2) that spans four oxidation states.^39^ We now report that replacing the Fe center in the [poat]^3−^ site with a Mn ion provides a new heterobimetallic system that can be oxidized to the Fe^III^Mn^IV^ level and serve as a synthetic model for FeMn-containing enzymatic active sites (Scheme 1).

## Results and discussion

### Preparative routes and structure

**Scheme 2:**
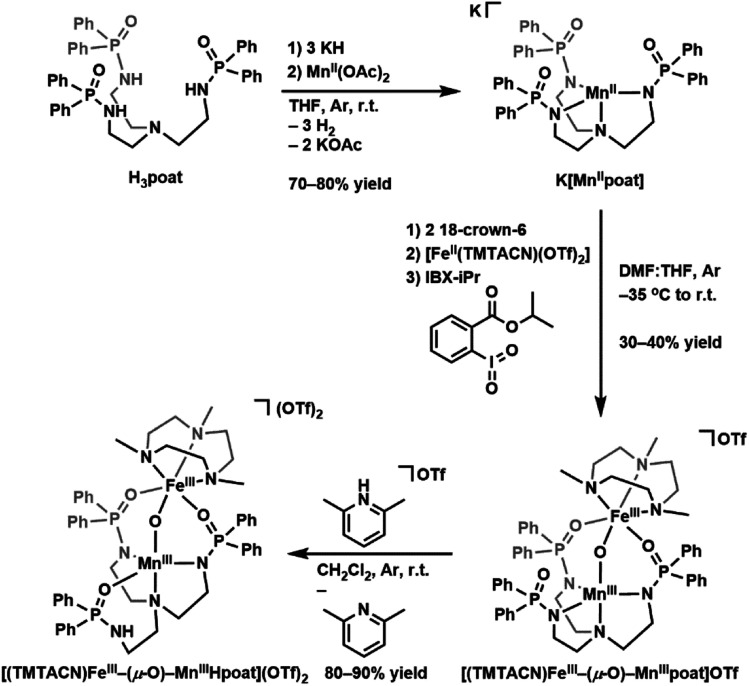
Preparative routes for K[Mn^II^poat], [(TMTACN)Fe^III^–(μ-O)–Mn^III^poat]OTf, and [(TMTACN)Fe^III^–(μ-O)–Mn^III^Hpoat](OTf)_2_.

**Fig. 1:**
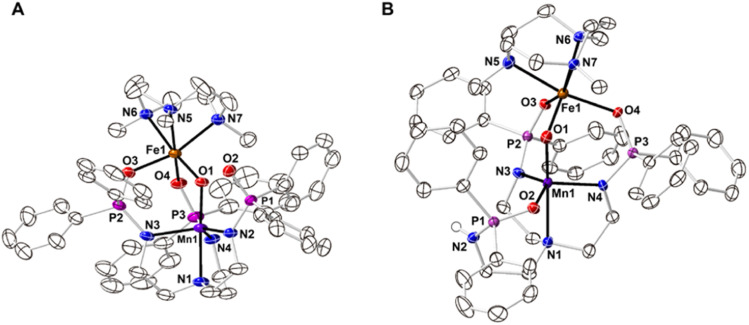
Thermal ellipsoid diagrams depicting the molecular structures of [Fe^III^(O)Mn^III^poat]^+^ (A) and [Fe^III^(O)Mn^III^Hpoat]^2+^ (B). Ellipsoids are drawn at the 50% probability level, and only the phosphinic amide H atom is shown for clarity. The triflate counterions are outer-sphere and are not interacting with the cation.

The starting Mn synthon, K[Mn^II^poat], was prepared by treating H_3_poat with three equivalents of KH and then subsequently adding Mn^II^(OAc)_2_ (Scheme 2). The isolated K[Mn^II^poat] salt, in the presence of 18-crown-6 and [Fe^II^(TMTACN)(OTf)_2_], was allowed to react with isopropyl 2-iodoxybenzoate at −35 °C to yield [(TMTACN)Fe^III^–(μ-O)–Mn^III^poat](OTf) ([Fe^III^(O)Mn^III^poat]OTf); crystalline needles suitable for X-ray diffraction were obtained after multiple rounds of recrystallization at room temperature. The molecular structure revealed an Fe–O–Mn core with Mn–O1 and Fe–O1 bond lengths of 1.767(3) and 1.802(3) Å, respectively, and an Fe⋯Mn distance of 3.205(5) Å (Fig. 1A and Table 1); these values are comparable with those of the di-Fe^III^ analog.^39^ The Mn^III^ site in the [poat]^3−^ framework adopts a trigonal bipyramidal geometry with an N_4_O primary coordination sphere comprising the N-atom donors of the [poat]^3−^ ligand and a bridging oxido ligand; the Fe^III^ site in the Fe-TMTACN adduct is 6-coordinated with an N_3_O_3_ primary coordination sphere comprising the TMTACN ligand, two O-atoms from the phosphinic amido groups from the [poat]^3−^ ligand, and the bridging oxido ligand. This heterobimetallic complex, with its three-atom-bridge motif, a dynamic primary coordination sphere, and the ability to form an intramolecular H-bond network, incorporates crucial elements observed in the FeMn active sites of Class Ic RNR and R2lox.^12,14,16,41^

**Table 1:** Selected bond lengths/distances (Å) and angles (^o^) for [Fe^III^(O)Mn^III^poat]^+^ and [Fe^III^(O)Mn^III^Hpoat]^2+^

	[Fe^III^(O)Mn^III^poat]^+^	[Fe^III^(O)Mn^III^Hpoat]^2+^
**Bond lengths/distances (Å)**		
Mn1–N1	2.071(3)	2.288(2)
Mn1–N2	2.008(3)	—
Mn1–O2	—	2.033(2)
Mn1–N3	2.079(4)	2.007(2)
Mn1–N4	2.058(4)	2.009(2)
Mn1–O1	1.767(3)	1.793(2)
Fe1–O1	1.802(3)	1.795(2)
Fe1–O3	2.026(3)	2.000(1)
Fe1–O4	2.046(3)	2.060(2)
Fe1–N5	2.219(3)	2.234(2)
Fe1–N6	2.261(3)	2.211(2)
Fe1–N7	2.225(4)	2.212(2)
Mn1⋯Fe1	3.205(4)	3.206(1)
av Mn1–N/O_eq_	2.048(3)	2.016(2)
av Fe1–N_TMTACN_	2.235(3)	2.219(2)
*d*[Mn1–N/O_eq_]^a^	0.301	0.244
*d*[Fe1–N_TMTACN_]^a^	1.524	1.499

**Angles (** ^**o**^ **)**		
O1–Mn1–N1	177.85(14)	178.25(7)
N2–Mn1–N3	120.53(15)	—
O2–Mn1–N3	—	133.29(7)
N3–Mn1–N4	103.63(15)	107.64(7)
N2–Mn1–N4	129.26(15)	—
O2–Mn1–N4	—	114.56(7)
Mn1–O1–Fe1	127.80(15)	126.62(8)
O3–Fe1–O4	96.08(12)	98.29(6)
N5–Fe1–N6	78.54(12)	79.89(7)
N5–Fe1–N7	79.00(13)	78.84(7)
N6–Fe1–N7	78.24(13)	79.29(7)

**Calculated values**		
*τ*_5_ b	0.810	0.749

a *d*[M–N/O_*x*_] denotes the displacement of the metal atom from the 3-atom plane. N/O_eq_ represented the plane formed by N2, N3, N4 or O2, N3, N4. N_TMTACN_ represents the plane formed by N5, N6, N7.

b Trigonality structural parameter, *τ*_5_ = (*β* − *α*)/60°. *β* is the largest bond angle observed, and *α* is the second largest bond angle observed.

The movement of protons is critical in many enzymatic processes,^4,42–46^ and so we explored the protonation/deprotonation steps within [Fe^III^(O)Mn^III^poat]^+^. The addition of one equivalent of 2,6-lutidinium triflate to [Fe^III^(O)Mn^III^poat]^+^ at −60 °C resulted in the immediate replacement of the *λ*_max_ = 795 nm feature with a new band at 750 nm (Scheme 1 and Fig. S1A†), which was determined from electron paramagnetic resonance (EPR) spectroscopy (see below) to be the protonated species [(TMTACN)Fe^III^–(μ-OH)–Mn^III^poat]^2+^ ([Fe^III^(OH)Mn^III^poat]^2+^). This hydroxido-bridged complex was thermally unstable, even at −60 °C, and further converted to a species having a peak at *λ*_max_ = 648 nm after one hour or upon warming to room temperature (Scheme 1 and Fig. S1B†). Isolation of the product at room temperature gave [(TMTACN)Fe^III^–(μ-O)–Mn^III^Hpoat](OTf)_2_ ([Fe^III^(O)Mn^III^Hpoat](OTf)_2_), whose structure revealed that the proton was transferred to the N-atom of one phosphinic amido group with its P=O unit now coordinated to the Mn^III^ center (Fig. 1B and Table 1).‡‡[Fe^III^(O)Mn^III^Hpoat](OTf)_2_ can also be prepared *via* a different stepwise route from K[Mn^II^poat] and [Fe^II^(TMTACN)(OTf)_2_]; for details, see ESI, Scheme S1 and Fig. S9.† The Mn–O1 and Fe–O1 bond lengths of 1.793(2) and 1.795(2) Å, respectively, and the Fe⋯Mn distance of 3.206(1) Å are similar to the measurements in the core of [Fe^III^(O)Mn^III^poat]^+^. The H-atom at the N-position could be identified using a difference-Fourier map and was also corroborated by a strong *ν*(N–H) feature at 3260 cm^−1^ in the FT-IR spectrum (Fig. S2†). The systematic blue-shifting of this optical transition upon protonation, as well as the intramolecular H^+^ transfer, were also observed for the di-Fe series.^39^

### Magnetic properties of the Mn^III^Fe^III^ complexes

**Fig. 2:**
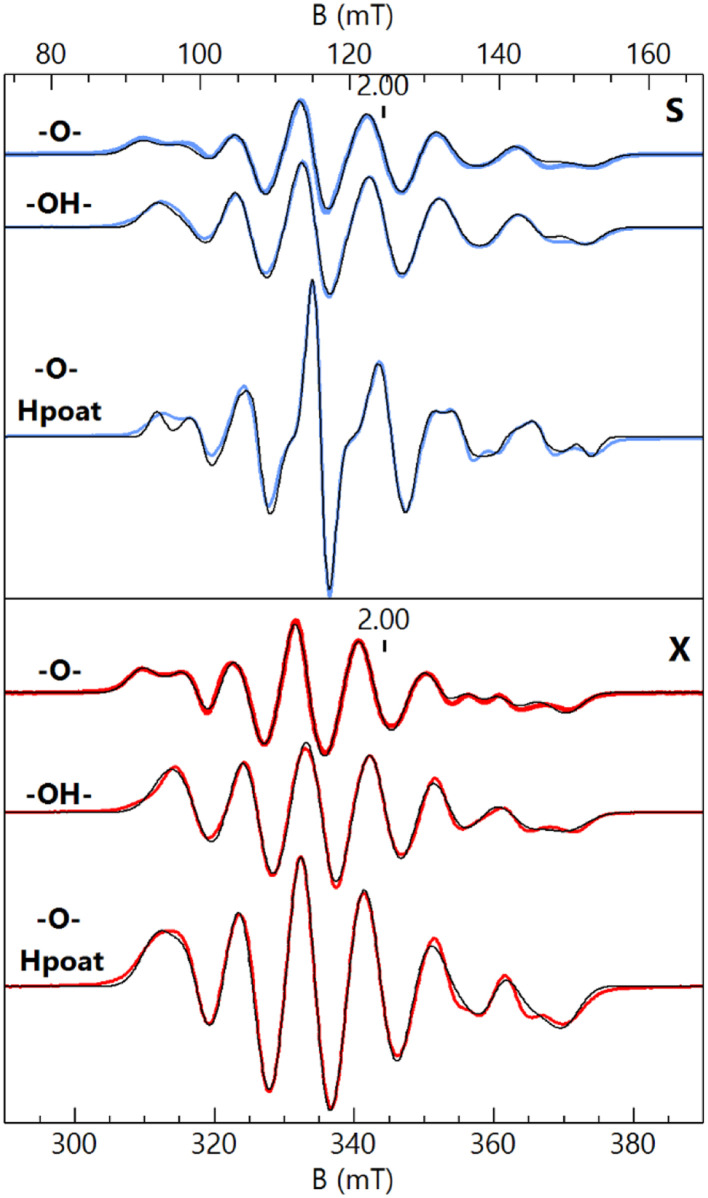
S- (blue traces, 3.485 GHz) and X-band (red traces, 9.636 GHz) EPR spectra of [Fe^III^(O)Mn^III^poat]^+^ (–O–), [Fe^III^(OH)Mn^III^poat]^2+^ (–OH–), and [Fe^III^(O)Mn^III^Hpoat]^2+^ (–O– Hpoat), 3 mM in CH_2_Cl_2_ (–O–, –OH–) or CH_2_Cl_2_:THF (–O– Hpoat). Measurement temperature, 20 K. The black traces are *S* = 1/2 simulations using the parameters given in Table 2. The *g* = 2 position is indicated.

S- and X-band EPR spectroscopy (Fig. 2) revealed that each Fe^III^Mn^III^ complex has a distinct six-line hyperfine feature from its ^55^Mn center near *g* = 2. Although the spectral differences between the complexes are subtle, they are reproducible and could be simulated with distinctly different parameters for each species. The hyperfine pattern at *g* = 2 is indicative of an *S* = 1/2 spin-coupled system containing an *S* = 5/2 Fe^III^ ion antiferromagnetically coupled to an *S* = 2 Mn^III^ ion.^6,17–19,32,33,47^ Simultaneous least-squares fitting of the spectra for both frequencies provided the *g*- and *A*(^55^Mn)-tensors for the coupled system (Table 2). A rotation between the *g*- and A-tensors was required to simulate both the S- and X-band spectra with the same parameter set. The *g*-values are all larger than 2 because the *g*-values of the coupled *S* = 1/2 state are given by *g*_c_ = 2 − (4/3)(*g*_Mn_ − 2) where the *g*-values for the Mn^III^ centers are all less than 2 assuming *g*_Fe_ = 2. The deviations in the Mn^III^*g*-values from 2 are at most 0.03, indicating that the spin–orbit contributions to the A-tensor are small. The spin-dipolar contribution to the A-tensor (**A**_**SD**_) was obtained by subtracting the isotropic value (A_iso_) = trace(**A**_**Mn**_)/3 from **A**_**Mn**_ for the uncoupled *S* = 2 Mn^III^ site. The uncoupled values were determined from **A**_Mn_ = −3/4 **A**^C^_Mn_, where **A**^C^_Mn_ is the magnetic hyperfine tensor for coupled spin *S* = 1/2. Only the magnitude of the hyperfine values can be determined from EPR spectroscopy, but the isotopic value is known to be negative. The A_iso_ values are near −200 MHz that are typical of Mn^III^ complexes.^6,18,19,32,33,48–50^ The values of **A**_**SD**_ are close to those obtained from density functional theory (DFT) calculations for the complexes and are within the uncertainty of the small spin–orbit contributions. The *g*- and A_SD_-tensors for the Mn^III^ sites are rhombic, owing to a distorted empty d_*z*^2^_ orbital having mixtures with other d-orbitals as indicated by DFT. The distortion is influenced by the Fe–O–Mn angle being substantially less than 180°. As we have previously described, a rhombic **A**_**SD**_ tensor was observed for the isoelectronic d^4^ Fe^IV^ site of [Fe^III^(O)Fe^IV^poat]^2+^, in which the di-Fe oxido bridge core is also bent; moreover, these findings contrast with those found for the related [Fe^IV^poat(O)]^−^ complex that has an axial **A**_**SD**_ tensor.^36,39^

**Table 2:** Parameters derived from EPR spectroscopy and DFT (in parentheses) for the Fe^III^Mn^III^ species^a^

Complex	*g*	**A**, *S* = 1/2	g ∠A *α*, *β*, *γ*^b^	A_iso_*S*_Mn_ = 2	**A**_SD,_ *S*_Mn_ = 2 (DFT A_SD_)	*J* c
[Fe^III^(O)Mn^III^poat]^+^	2.042	215	73	−200	39, 17, −57 (51, 11, −62)	120 (170)
	2.031	243	54			
	2.007	341	76			
[Fe^III^(OH)Mn^III^poat]^2+^	2.026	209	90	−197	41, 5, −46 (43, 10, −53)	40 (40)
	2.024	255	27			
	2.007	323	62			
[Fe^III^(O)Mn^III^Hpoat]^2+^	2.037	202	77	−196	44, 6, −50 (40, 25, −65)	100 (125)
	2.025	254	53			
	2.008	327	71			

a A-values are in MHz.

b Euler angles *α*, *β*, *γ* indicate the rotation of **A** relative to ***g***.

c cm^−1^ (*J***S**_**Fe**_**·S**_**Mn**_).

**Fig. 3:**
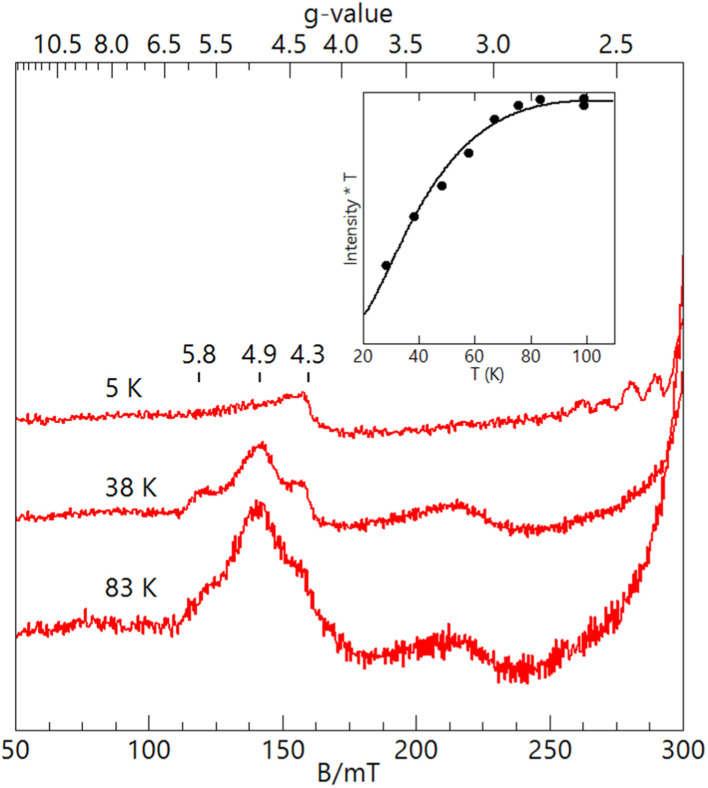
X-band (9.620 GHz) EPR spectra of [Fe^III^(OH)Mn^III^poat]^2+^ at 3 mM in CH_2_Cl_2_ at the measurement temperatures listed. The inset shows the temperature dependence of the *S* = 3/2 signal fitted with *J* = 40 cm^−1^ (*J***S**_**Fe**_**·S**_**Mn**_). The signal at *g* = 4.3 is from a minor Fe^III^ impurity.

The EPR features of [Fe^III^(OH)Mn^III^poat]^2+^ were further examined at higher temperatures to demonstrate the presence of the hydroxido bridging ligand. Upon warming above 20 K, a new signal appeared with features at *g* = 5.8, 4.9, and 3.0, which was absent from both [Fe^III^–(μ-O)–Mn^III^] complexes (Fig. 3). These *g*-values are expected for an *S* = 3/2 spin system with *E*/*D* = 0.16. When the spectra were plotted as intensity × temperature, an intensity increase was found at higher temperature as the *S* = 3/2 excited state became populated. The temperature dependence of the *S* = 3/2 signal (Fig. 3, inset) was fitted using the spin Hamiltonian *J***S**_**Fe**_**·S**_**Mn**_ with *J* = +40 cm^−1^, a value indicative of an antiferromagnetically coupled dinuclear system with a hydroxido bridging ligand.^51^ DFT calculations of the [Fe^III^(OH)Mn^III^poat]^2+^ complex also gave a *J*-value of +40 cm^−1^. The zero-field values *D* for the sites cannot be determined but are ∼0.5 cm^−1^ for Fe^III^TACN complexes and ∼2 cm^−1^ for Mn^III^ complexes in a trianionic ligand scaffold similar to [poat]^3−^.^49^ These values are small relative to *J* = 40 cm^−1^ and have only a minor effect (only a few percent) on the observed A-tensor.

For [Fe^III^(O)Mn^III^poat]^+^ and [Fe^III^(O)Mn^III^Hpoat]^2+^, the temperature dependence of the microwave power required to half-saturate the EPR signal (*P*_1/2_) was measured over the temperature range 4–40 K (Fig. S3†). A fit of the data (Fig. S3†) to an Orbach relaxation curve and DFT gave the experimental and computed *J*-values, respectively (Table 2). The values are greater than 100 cm^−1^ and are consistent with an oxido bridging ligand.^51^ These values are similar to those of the oxido bridged Fe^III^Mn^III^ complexes with TACN and TMTACN ligands (*J* ∼ 130 cm^−1^) from magnetic susceptibility measurements.^31^

**Fig. 4:**
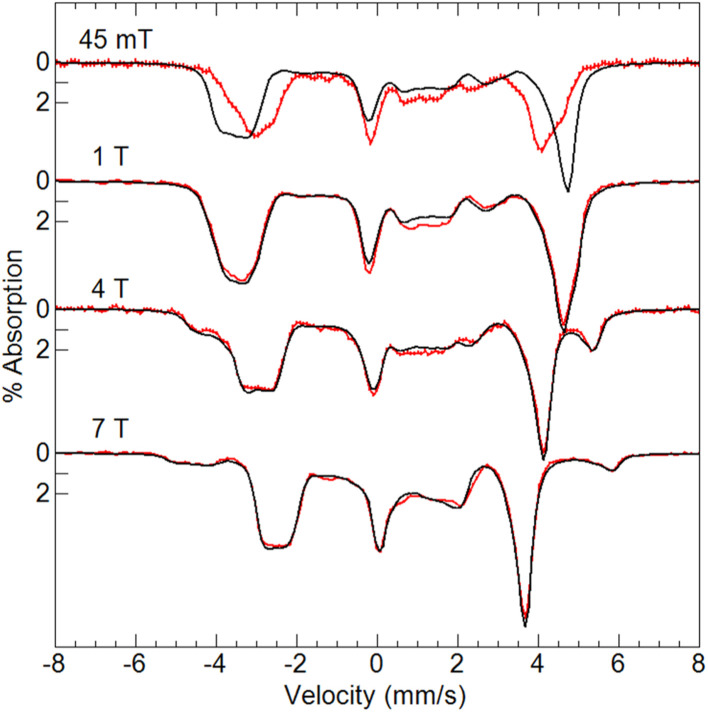
Mössbauer spectra (red traces) of [^57^Fe^III^(O)Mn^III^poat]^+^ in PrCN at 4.2 K and the magnetic fields listed. The simulations (black traces) are for *S* = 5/2 Fe^III^ antiferromagnetically exchange-coupled to *S* = 2 Mn^III^ (see text for parameters).

Variable-field ^57^Fe Mössbauer spectra and simulations of [Fe^III^(O)Mn^III^poat]^+^ (Fig. 4) are indicative of an *S* = 5/2 Fe^III^ center exchange-coupled antiferromagnetically to an *S* = 2 Mn^III^ center with *J* > 100 cm^−1^, thus producing a coupled *S* = 1/2 ground state. The Fe^III^ site has an isomer shift (*δ*) of 0.53 mm s^−1^, quadrupole splitting (Δ*E*_Q_) of −1.84 mm s^−1^, and an isotropic A-tensor of −20 T. These parameters are indicative of a high-spin Fe^III^ center.^7,47^ However, the experimental and simulated ^57^Fe Mössbauer spectra did not match at low magnetic field (45 mT, Fig. 4). The cause of this mismatch appears to result from the spin expectation of the Fe being reduced from its normal value to give an overall spectral line splitting that was smaller than expected. One possibility is that this lower spin expectation occurs for coupled metal spin systems with zero-field splitting (*D*) comparable to the exchange coupling (*J*). However, this possibility would cause the simulations at higher fields to not match the data, which they clearly do (Fig. 4), and was therefore ruled out.

The origin of the effect was discovered to be from the hyperfine interaction of the ^55^Mn nuclear spin (*I* = 5/2) with the electronic system spin (*S* = 1/2). For Fe complexes, the energy of the hyperfine interaction (**S**·**A**_**Fe**_·**I**_**Fe**_) is approximately 0.001 cm^−1^. For small magnetic fields produced by permanent magnets (∼50 mT) that are commonly used in Mössbauer spectrometers, the energy splitting between the electronic spin states is ∼0.04 cm^−1^, consequently, the electronic spin expectation at the Fe site is dominated by the magnetic field interaction (m_B_**S**·**g**·**B**). The EPR analysis of the *S* = 1/2 signal in [Fe^III^(O)Mn^III^poat]^+^ (Table 2) gave an isotropic hyperfine constant for ^55^Mn of *A*_iso_ = 260 MHz (0.0087 cm^−1^), which is more than 10% of the electronic energy splitting. Thus, the nuclear spin of Mn site significantly alters the electronic spin expectation observed at the Fe site.

**Fig. 5:**
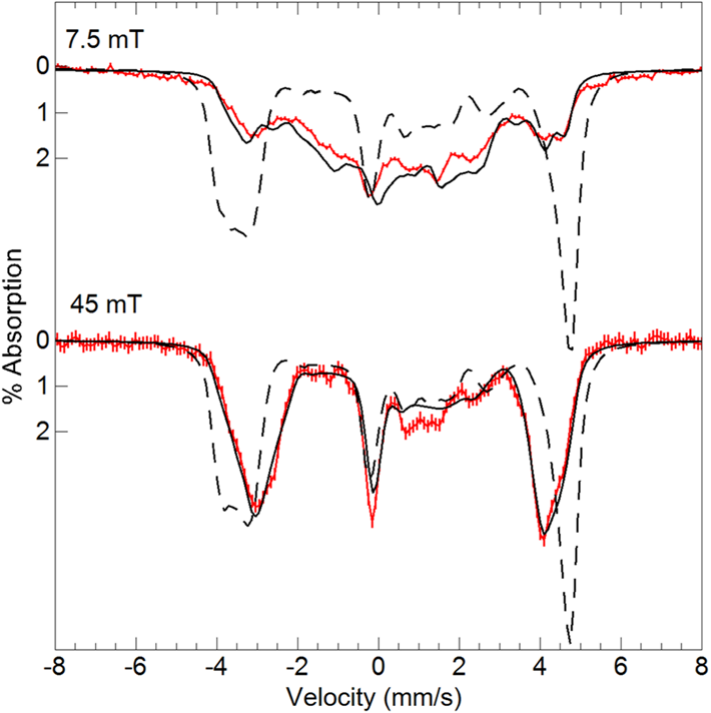
Mössbauer spectra (red traces) of [^57^Fe^III^(O)Mn^III^poat]^+^ in PrCN at 4.2 K and the magnetic fields listed. The simulations (black traces) are for *S* = 5/2 Fe^III^ antiferromagnetically exchange-coupled to *S* = 2 Mn^III^ with inclusion of the ^55^Mn hyperfine interaction using the A-tensor and rotation derived from EPR spectroscopy (Table 2). The dashed lines are without the ^55^Mn hyperfine interaction.

To examine how this interaction affects the analysis of the Mössbauer data, we have incorporated the ^55^Mn hyperfine interaction (**S**·**A**_**Mn**_·**I**_**Mn**_) into our simulation software to allow diagonalization of the spin Hamiltonian with the electronic and Mn nuclear spin states. The new simulations of Mössbauer spectra collected at 7.5 and 45 mT show a dramatic change which closely matches the data (Fig. 5). The effect of this interaction is only observed at lower magnetic fields, while simulations at higher fields are unaffected by the Mn nuclear spin because the electronic spin at the Fe site is dominated by the magnetic field interaction.

### Preparation and properties of an Fe^III^–(μ-O)–Mn^IV^ complex

**Fig. 6:**
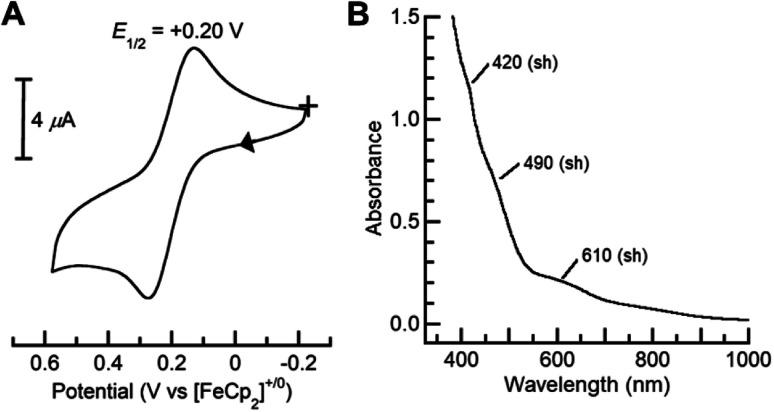
Cyclic voltammogram (A) of [Fe^III^(O)Mn^III^poat]^+^ collected at 100 mV s^−1^ in CH_2_Cl_2_, and electronic absorption spectrum of [Fe^III^(O)Mn^IV^poat]^2+^ (B) measured in a 0.20 mM CH_2_Cl_2_ solution at −60 °C.

Dioxygen-activating FeMn enzymes such as RNR Class Ic and R2lox have been proposed to access higher oxidation levels (*e.g.*, Fe^III^Mn^IV^ and Fe^IV^Mn^IV^) to achieve function;^6–9,12,14,18^ we therefore used cyclic voltammetry to evaluate the redox properties of our Fe^III^Mn^III^ complexes. The cyclic voltammogram of [Fe^III^(O)Mn^III^poat]^+^ revealed a nearly reversible, one-electron redox event at +0.20 V *vs.* [FeCp_2_]^+/0^, which was assigned to the Fe^III^Mn^IV/III^ process (Fig. 6A). This potential is significantly more negative than the Fe^III^Fe^IV/III^ potential of +0.55 V *vs.* [FeCp_2_]^+/0^ observed in the di-Fe analog,^39^ suggesting that the redox change occurs at the Mn site. Treatment of [Fe^III^(O)Mn^III^poat]^+^ with [FeCp(C_5_H_4_C(O)Me)]OTf or [N(*p*-C_6_H_4_Me)_3_]OTf at −60 °C resulted in the loss of the diagnostic peak at *λ*_max_ = 795 nm and the concurrent appearance of shoulders at 420, 490, and 610 nm (Fig. 6B). While these features are poorly resolved, they resemble those reported for the catalytic Fe^III^Mn^IV^ intermediate in RNR Ic.^12^ This new species did not show an EPR signal, suggesting an integer spin complex (Fig. S4†). This redox process was reversible: the addition of FeCp_2_ to this new compound regenerated the optical and EPR features of [Fe^III^(O)Mn^III^poat]^+^ and [FeCp_2_]^+^, supporting the hypothesis that the reaction was an outer-sphere electron transfer.

**Fig. 7:**
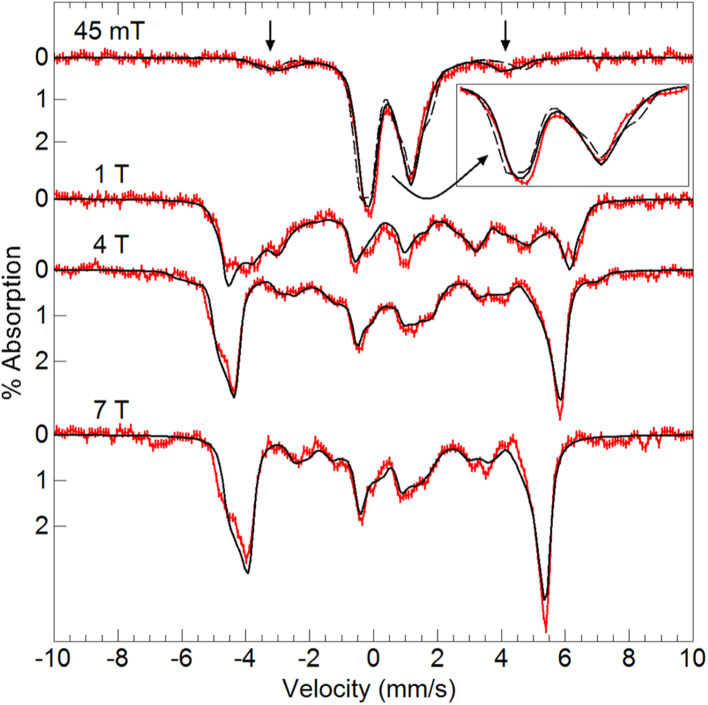
Mössbauer spectra (red traces) of [^57^Fe^III^(O)Mn^IV^poat]^2+^ in PrCN at 4.2 K and the magnetic fields listed. The simulations (black traces) are for an *S* = 5/2 Fe^III^ ion antiferromagnetically exchange-coupled to *S* = 3/2 Mn^IV^ ion (see text for parameters). The vertical arrows mark spectral features from a minor amount of [Fe^III^(O)Mn^III^poat]^+^. The dashed line in the 45 mT spectrum is without the ^55^Mn hyperfine interaction (see inset).

Variable-field ^57^Fe Mössbauer spectra and simulations of the oxidized species showed that the features associated with [Fe^III^(O)Mn^III^poat]^+^ are nearly absent (Fig. 7). The simulations were based on an *S* = 5/2 Fe^III^ ion antiferromagnetically exchange-coupled to an *S* = 3/2 Mn^IV^ ion with *J* > 100 cm^−1^, producing a coupled system with an *S* = 1 state that is lowest in energy. The Fe^III^ site has the parameters *δ* = 0.50 mm s^−1^, Δ*E*_Q_ = −1.31 mm s^−1^, and an isotropic **A**-tensor of −20 T. These values are typical of a high-spin Fe^III^ center and close to those of [Fe^III^(O)Mn^III^poat]^+^, indicating that the Mn center has been oxidized. The new species was therefore formulated as [(TMTACN)Fe^III^–(μ-O)–Mn^IV^poat]^2+^ ([Fe^III^(O)Mn^IV^poat]^2+^). Although this species has an *S* = 1 spin state, no signals were observed in parallel-mode EPR spectra. A minor species, assigned to [Fe^III^(O)Mn^III^poat]^+^, was also present in the Mössbauer spectra (15%) and is most evident in the 45 mT spectrum – it was included in all simulations. As observed in the spectra of [Fe^III^(O)Mn^III^poat]^+^, the Mössbauer spectrum of [Fe^III^(O)Mn^IV^poat]^2+^ at 45 mT was affected by the Mn nuclear spin (Fig. 7, dashed line). The effect is minor owing to the small spin expectation for the spin *S* = 1 state at low field (Fig. 7, inset).

There are several examples of complexes with FeMn centers, and in each, the oxidation states of the metal centers are below 4+.^23–29,31–34^ Our knowledge of synthetic systems containing an Fe^III^–(μ-O)–Mn^IV^ core has been limited to a single previous report of one complex whose spectroscopic and magnetic characterizations are incomplete.^31^ In fact, the best-studied system prior to [Fe^III^(O)Mn^IV^poat]^2+^ is RNR Ic R2-X,^6,7,12^ the active intermediate that initiates long-range PCET from the R1 subunit for catalytic function. Comparison of the spectroscopic properties of these two Fe^III^Mn^IV^ systems revealed several common features: similar electronic absorption spectra, Mössbauer parameters, and *J*-values (Table 3). As the PCET pathway between the R1/R2 subunits involves tyrosine residues, we tested the reactivity of [Fe^III^(O)Mn^IV^poat]^2+^ with various *para*-substituted phenols, such as 4-MeO-PhOH (BDFE = 79.9 kcal mol^−1^),^52^ 4-*tert*-butyl-PhOH (83.5),^53^ and 4-F-PhOH (83.8),^52^ at −60 °C in CH_2_Cl_2_ (Scheme 1 and Fig. S5–S8†). For instance, upon treatment with an excess (≥5 equivalents) of 4-MeO-PhOH, the optical and EPR features of the Fe^III^Mn^IV^ species were replaced by those of [Fe^III^(OH)Mn^III^poat]^2+^ in a near quantitative amount (>90% by EPR quantification; Fig. S5†); upon warming, the Fe^III^–(μ-OH)–Mn^III^ core underwent intramolecular proton transfer to form [Fe^III^(OH)Mn^III^poat]^2+^, analogous to the rearrangement observed after the protonation of [Fe^III^(O)Mn^III^poat]^+^ (Scheme 1). The organic product was identified to be 5,5′-dimethoxy-[1,1′-biphenyl]-2,2′-diol (246.2 *m*/*z*; > 95% by GC-MS quantification; Fig. S6†), which is consistent with a PCET mechanism in which the *para*-substituted phenol reacts with [Fe^III^(O)Mn^IV^poat]^2+^ to first form the phenoxyl radical, which then undergoes a bimolecular homocoupling to produce the bisphenol.^39,54^ These results demonstrate for the first time that systems with an Fe^III^–(μ-O)–Mn^IV^ core are indeed competent to perform this type of PCET process.

**Table 3:** Spectroscopic comparison of [Fe^III^(O)Mn^IV^poat]^2+^ and Fe/Mn RNR

Complex	*S*_T_	*λ* (nm, *ε*_M_)	^57^Fe *δ*/Δ*E*_Q_^a^	*A*_*x*,*y*,*z*_(^57^Fe)^b^	*J* c
[Fe^III^(O)Mn^IV^poat]^2+^	1	420 (sh)	0.50/–1.31	−20	>100^d^
		490 (sh)		−20	
		610 (sh)		−20	
RNR Ic R2-X^e^	1	360 (sh)	0.52/|1.32|	−23.0	200
		408 (sh)		−22.2	
		477 (1500)		−21.7	

a mm s^−1^.

b Tesla.

c cm^−1^.

d From Mössbauer spectroscopy using +*J***S**_**Fe**_**·S**_**Mn**_.

e Ref. 6, 7 and 12.

Our reactivity studies additionally found that [Fe^III^(O)Mn^IV^poat]^2+^ did not react with compounds having relatively weak C–H bonds, such as 9,10-dihydroanthracene (72.9) and xanthene (70.2). This lack of reactivity was also found for the analogous [Fe^III^(O)Fe^IV^poat]^2+^ species, which also reacts with similar phenols to first produce [Fe^III^(OH)Fe^III^poat]^2+^.^39^ In both systems, the homolytic cleavage of the O–H bond of the phenolic substrate leads to the initial protonation of the oxido ligand, which is sterically hindered by the [poat]^3−^ ligand and methyl groups of the TMTACN ligand. We proposed that the PCET process involved for homolytic ArO–H bond cleavage by [Fe^III^(O)Fe^IV^poat]^2+^ favors the protonation of a site near to the metal center even though it was hindered (that is, rather than protonating the ligand to form [Fe^III^(O)Fe^III^Hpoat]^2+^). A similar process appears to be operative for the Fe^III^Mn^IV^ analog. However, [Fe^III^(O)Mn^IV^poat]^2+^ did not react with 2,6-*tert*-butyl-4-R-PhOH (R = –OMe (BDFE = 72.6 kcal mol^−1^), –^*t*^Bu (75.5), and –H (77.0),^52^ substrates that are substantially more sterically hindered. Steric effects cannot be the only reason for this lack of reactivity because we have previously shown that the analogous [Fe^III^(O)Fe^IV^poat]^2+^ did react with these substrates.^39^ We do not completely understand the lack of reactivity for [Fe^III^(O)Mn^IV^poat]^2+^ and its apparent difference from that found for the Fe^III^Fe^IV^ analog. With this said, the two bimetallic complexes have differing Fe^III^M^IV^/Fe^III^M^III^ redox potentials with [Fe^III^(O)Fe^IV^poat]^2+^ being the stronger oxidant that may contribute to its increased reactivity.^39^

## Conclusions

In summary, the rigid [poat]^3−^/TMTACN ligand scaffold allowed us to construct discrete FeMn complexes and examine their individual or coupled proton and electron transfer steps. We described the reactivity, spectroscopic character, and electrochemical properties of a high-valent compound that contains an Fe^III^–(μ-O)–Mn^IV^ core. We have previously shown that mononuclear Mn–O(H) complexes in trigonal symmetry have lower M^IV/III^ reduction potentials than their Fe counterparts,^55–57^ but, to our best knowledge, this work presents the first direct experimental comparison of the redox properties, and the resultant changes in the electronic structures, of related FeMn and di-Fe compounds at an oxidation state above 3+. Our studies found that the lower potentials of FeMn complexes compared to their di-Fe analog could be used to form a complex with an Fe^III^–(μ-O)–Mn^IV^ core. Within a biological context, a more thermodynamically accessible Mn^IV^ ion over an Fe^IV^ ion can be one important contributing factor leading to the selective binding of FeMn sites in Class Ic RNR and R2lox enzymes, especially when the key catalytic steps do not require an overly potent oxidant (such as di-Fe^IV^) that may cause undesirable damage to the proteins. [Fe^III^(O)Mn^IV^poat]^2+^ shares similar electronic, magnetic, and spin-exchange features as RNR R2 Ic-X, suggesting the former to be the first well-characterized model for the biological Fe^III^Mn^IV^ core.

The Mössbauer findings on [Fe^III^(O)Mn^IV^poat]^2+^ and [Fe^III^(O)Mn^III^poat]^+^ revealed a mismatch between the data and simulations at low external magnetic fields that is caused by the hyperfine interaction of the ^55^Mn nuclear spin with the electronic system spin. This effect on the Mössbauer spectra of inorganic complexes is uncommon because it requires an electronic interaction between Fe and a second metal ion having a nuclear spin and large hyperfine values. The possible influence of the ^55^Mn nuclear spin has been suggested in the Mössbauer spectra of an Mn^IV^Fe^IV^ intermediate in *Chlamydia trachomatis* ribonucleotide reductase.^8^ Our findings for [Fe^III^(O)Mn^III^poat]^+^ and [Fe^III^(O)Mn^IV^poat]^2+^ support this premise and demonstrate a new analysis to obtain quantitative agreement between Mössbauer data and simulations that incorporate the ^55^Mn A-tensor and rotation derived from EPR spectroscopy. To our knowledge, this is the first quantitative demonstration of the effect of the hyperfine interaction from a non-Fe nucleus on the Mössbauer spectra of inorganic complexes. Our work also highlights the importance of accessibility during PCET processes: the initial formation of the [Fe^III^(OH)Mn^III^poat]^2+^ species upon PCET with phenolic substrates, in which the proton transfers to the bridging oxido ligand and the electron reduces the Mn^IV^ center, appears to be necessary for reactivity to occur. Within a growing body of literature and improved understanding of FeMn enzymes and their oxidative chemistry, the [(TMTACN)Fe^*m*^–(μ-O(H))–Mn^*n*^(H)poat]^*z*+^ system serves as a useful structural, spectroscopic, and functional model to complement biochemical investigations.

## Data availability

The datasets supporting this article have been uploaded as part of the ESI.†

## Author contributions

The concept and experimental studies were performed by J. L. L. and A. S. B. The magnetic measurements and DFT calculations were performed by S. B., E. L. B., and M. P. H. The crystallographic measurements and analyses were performed by J. W. Z. All of the authors actively participated in manuscript preparation and editing.

## Conflicts of interest

There are no conflicts to declare.

## Supplementary Material

SC-015-D3SC04900K-s001

SC-015-D3SC04900K-s002
