# Supplementary material for: Accessing a synthetic FeIIIMnIV core to model biological heterobimetallic active sites
Source: Chem Sci. 2023 Dec 27;15(8):2817–26. doi: 10.1039/d3sc04900k (PMC10882444; doi:10.1039/d3sc04900k)
Supplement: SC-015-D3SC04900K-s001 [file SC-015-D3SC04900K-s001.pdf]

Electronic Supplementary Material (ESI) for Chemical Science.
This journal is © The Royal Society of Chemistry 2023

Electronic Supplementary Information for

Accessing a Synthetic $Fe^{III}Mn^{IV}$ Core to Model Biological Heterobimetallic Active Sites

Justin L. Lee,[§] Saborni Biswas,[⊥] Joseph W. Ziller,[§] Emile L. Bominaar,[⊥] Michael P. Hendrich,[⊥] and A. S. Borovik*[§]

[§]Department of Chemistry, University of California-Irvine, 1102 Natural Sciences II, Irvine, California 92697, United States

[⊥]Department of Chemistry, Carnegie Mellon University, 4400 Fifth Avenue, Pittsburgh, Pennsylvania 15213, United States

*Email: aborovik@uci.edu

Contents

General methods.	S3
Physical methods.	S3
Computational studies.	S4
Crystallography.	S6
Preparation of K[Mn^{II}poat].	S9
Preparation of [(TMTACN)Fe^{III}–(μ-O)–Mn^{III}poat]OTf.	S9
Preparation of K[Mn^{III}poat(OH)].	S9
Preparation of [(TMTACN)Fe^{II}–(μ-OH)–Mn^{III}poat]OTf.	S10
Preparation of [(TMTACN)Fe^{III}–(μ-O)–Mn^{III}Hpoat]$(OTf)_2$.	S11
Low-temperature UV-vis solution studies.	S12
Preparation of EPR solution samples at low-temperature.	S12
Preparation of Mössbauer solution samples at room-temperature.	S13
Preparation of Mössbauer solution samples at low-temperature.	S13

GC-MS experiments. S13

Scheme S1. Preparative routes for $K[Mn^{III}poat(OH)]$, $[Fe^{II}(OH)Mn^{III}poat]OTf$, and $[Fe^{III}(O)Mn^{III}Hpoat](OTf)_2$. S14

Figure S1. Electronic absorbance spectra of the protonation of $[Fe^{III}(O)Mn^{III}poat]^+$ to generate $[Fe^{III}(OH)Mn^{III}poat]^{2+}$ at low temperature and subsequent conversion to $[Fe^{III}(O)Mn^{III}Hpoat]^{2+}$. S15

Figure S2. FT-IR spectra of $[Fe^{III}(O)Mn^{III}poat]^+$ and $[Fe^{III}(O)Mn^{III}Hpoat]^{2+}$. S15

Figure S3. Orbach relaxation curve: microwave power of the EPR signal of $[Fe^{III}(O)Mn^{III}poat]^+$ versus temperature. S16

Figure S4. EPR spectrum of the oxidation of $[Fe^{III}(O)Mn^{III}poat]^+$ to $[Fe^{III}(O)Mn^{IV}poat]^{2+}$. S16

Figure S5. Electronic absorbance and EPR spectra of the reaction of $[Fe^{III}(O)Mn^{IV}poat]^{2+}$ towards 4-MeO-PhOH. S17

Figure S6. GC-MS plots of the reaction of $[Fe^{III}(O)Mn^{IV}poat]^{2+}$ towards 4-MeO-PhOH. S17

Figure S7. Electronic absorbance and EPR spectra of the reaction of $[Fe^{III}(O)Mn^{IV}poat]^{2+}$ towards 4-^{t}Bu-PhOH. S18

Figure S8. Electronic absorbance spectra of the reaction of $[Fe^{III}(O)Mn^{IV}poat]^{2+}$ towards 4-F-PhOH. S18

Figure S9. Thermal ellipsoid diagram depicting the molecular structure of $[Fe^{II}(OH)Mn^{III}poat]^+$. S19

Figure S10. DFT optimized structure of $[(TMTACN)Fe^{III}–(\mu\text{-O})–Mn^{III}poat]^+$ in BS state. S19

Figure S11. DFT optimized structure of $[(TMTACN)Fe^{III}–(\mu\text{-OH})–Mn^{III}poat]^{2+}$ in BS state. S20

Figure S12. DFT optimized structure of $[(TMTACN)Fe^{III}–(\mu\text{-O})–Mn^{III}Hpoat]^{2+}$ in BS state. S20

Figure S13. DFT optimized structure of $[(TMTACN)Fe^{III}–(\mu\text{-O})–Mn^{IV}poat]^{2+}$ in BS state. S21

Table S1. Selected bond lengths/distances (Å) and angles (°) for $[Fe^{II}(OH)Mn^{III}poat]^+$. S22

Table S2. Crystallographic data for $[Fe^{III}(O)Mn^{III}poat]^+$, $[Fe^{III}(O)Mn^{III}Hpoat]^{2+}$, and $[Fe^{II}(OH)Mn^{III}poat]^+$. S23

Table S3. $[(TMTACN)Fe^{III}–(\mu\text{-O})–Mn^{III}poat]^+$, BS (5/2, –2) optimized. S24

Table S4. $[(TMTACN)Fe^{III}–(\mu\text{-O})–Mn^{III}poat]^+$, F (5/2, 2) optimized. S27

Table S5. $[(TMTACN)Fe^{III}–(\mu\text{-OH})–Mn^{III}poat]^{2+}$, BS (5/2, –2) optimized. S30

Table S6. $[(TMTACN)Fe^{III}–(\mu\text{-OH})–Mn^{III}poat]^{2+}$, F (5/2, 2) optimized. S33

Table S7. [(TMTACN)Fe^{III}–(μ-O)–Mn^{III}Hpoat]$^{2+}$, BS (5/2, –2) optimized. S36

Table S8. [(TMTACN)Fe^{III}–(μ-O)–Mn^{III}Hpoat]$^{2+}$, F (5/2, 2) optimized. S39

Table S9. [(TMTACN)Fe^{III}–(μ-O)–Mn^{IV}poat]$^{2+}$, BS (5/2, –3/2) optimized. S42

Table S10. [(TMTACN)Fe^{III}–(μ-O)–Mn^{IV}poat]$^{2+}$, F (5/2, 3/2) optimized. S45

Table S11. Selected structure parameters for the BS/F state and *J* values of [(TMTACN)Fe^{III}–(μ-O{H})–$Mn^{III/IV}${H}poat]$^{+/2+}$ complexes. S48

References. S49

General Methods. All reagents were purchased from commercial sources and used as received unless otherwise noted. Solvents were sparged with argon and purified using a JC Meyer Co. solvent purification system with columns containing Q-5 and molecular sieves. Butyronitrile (PrCN) was purified according to a literature procedure.[1] Potassium hydride (KH) as a 30 % dispersion in mineral oil was filtered with a medium porosity glass frit and washed 5 times each with pentane and diethyl ether (Et_2O). Solid KH was dried under vacuum and stored under an inert atmosphere. Tetrabutylammonium hexafluorophosphate (TBAP) was recrystallized from hot EtOH. Ferrocene and 4-MeO-phenol were purified by sublimation. Organic syntheses, unless otherwise noted, did not require the use of an inert atmosphere. All inorganic syntheses and manipulations were performed under an argon atmosphere in a Vacuum Atmosphere Co. dry box. H_3poat,[2] [Fe^{II}(TMTACN)$(OTf)_2$],[3] [$FeCp_2$]OTf,[4] IBX-iPr,[5,6] 2,6-lutidinium triflate,[7] [FeCp(C_5H_4C(O)Me)]OTf,[4] and [N(*p*-C_6H_4Me)$_3$]OTf[4] were synthesized according to previous reports. The ^{57}Fe-enriched complexes were prepared using [$^{57}Fe^{II}$(TMTACN)$(OTf)_2$], which was synthesized using the $^{57}Fe^{II}Cl_2$ and $^{57}Fe^{II}(OTf)_2 \cdot 2CH_3CN$ precursors.[8]

Physical Methods. Room temperature electronic absorbance spectra were recorded with a Cary 50 using a 1.00 cm quartz cuvette. Low temperature electronic absorbance spectra were recorded on an Agilent UV-visible (UV-vis) spectrophotometer equipped with a Unisoku Unispeks cryostat in a 1.00 cm quarts cuvette. Solid-state Fourier transform infrared (FT-IR) spectra were collected on a Thermo Scientific Nicolet iS5 FT-IR spectrometer equipped with an iD5 ATR accessory. Cyclic voltametric

experiments were conducted using a CHI600G electrochemical analyzer. A 2.0 mm glassy carbon electrode was used as the working electrode at scan velocities between 2 and 2000 mV s^{-1}. A ferrocenium/ferrocene ($[FeCp_2]^{+/0}$) was used as an internal reference to monitor the reference electrode (Ag^+/Ag). Tetrabutylammonium hexafluorophosphate (TBAP) was used as the supporting electrolyte at a concentration of 0.1 M in CH_2Cl_2 or PrCN. Elemental analyses were performed on a Perkin-Elmer 2400 Series II CHNS elemental analyzer. ^{1}H–NMR spectra was collected on a Bruker CRYO500 spectrometer (500 MHz). Organic products were detected by gas-chromatography mass spectrometry (GC-MS) in the Mass Spectrometry Facility at the University of California, Irvine. The GC-MS was a Trace 1300 Gas Chromatograph from Thermo Scientific using a 15 m long x 0.25 mm i.d. TG-SQC column. The oven was held at 40 °C for 1 minute then heated at a rate of 20 °C min^{-1} to 290 °C and held for an additional 2 minutes. The mass spectrometry used electron ionization (70 eV) scanning (0.2/sec) from m/z 30 – 650.

X-band and S-band EPR spectra were recorded on a Bruker ELEXSYS spectrometer equipped with Oxford liquid helium cryostats. The quantification of all signals was measured relative to a CuEDTA spin standard prepared from a copper atomic absorption standard (Sigma-Aldrich). The microwave frequency was calibrated with a frequency counter and the magnetic field with an NMR gaussmeter. The sample temperature was calibrated against a calibrated cernox sensor (Lakeshore CX-1050) mounted inside an EPR tube. A modulation frequency of 100 kHz was used for all EPR spectra. Mössbauer spectra were recorded on either a variable field or a weak-field spectrometer operating in a constant acceleration mode in a transmission geometry using Janis Research Inc. cryostats that allow for a variation in temperature from 4 to 300 K. One of the dewars housed a superconducting magnet that allowed for the application of magnetic fields up to 7 T parallel to the γ-radiation. Isomer shifts are reported relative to Fe metal at 298 K. The simulation software *SpinCount* was written by one of the authors.[9] The software uses the spin Hamiltonian:

$$H = \boldsymbol{\beta S\cdot g\cdot B + S\cdot A_{Mn}\cdot I_{Mn} + S\cdot A_{Fe}\cdot I_{Fe}} - g_n\beta_n \mathbf{B\cdot I_{Fe}} + \mathbf{I_{Fe}\cdot P\cdot I_{Fe}}$$

$$\mathbf{I_{Fe}\cdot P\cdot I_{Fe}} = (eQV_{zz}/12)[3I_z^2 - I(I+1) + \eta(I_x^2 - I_y^2)]$$

where the parameters have the usual definitions and performs least-squares fitting of simulations to the spectra. For the Mössbauer spectral simulations which include the ^{55}Mn hyperfine interaction, the program diagonalizes the electronic and Mn hyperfine terms. The quantitative EPR simulations are generated with consideration of all intensity factors, which allows computation of simulated spectra for a specified sample concentration.

Computational Studies. The density functional theory (DFT) calculations were performed with the hybrid density functional B3LYP and the triple ζ basis sets TZVP at the P atoms and 6-311G at all other atoms, using the program suite Gaussian 09 revision D.01. The geometries have been optimized for both the ferromagnetic (F) and broken symmetry (BS) states in gas phase. The Cartesian coordinates of the optimized geometries have been listed in Tables S3-S10 and cartoons of the geometries for the BS mimics of the antiferromagnetic ground states are shown in Figures S10-S13. The BS calculations used the fragment approach implemented in Gaussian 09, with the Fe(TMTACN), Mn(poat), and the bridging O(H) moieties as fragments. For each complex, the DFT energies for the optimized structures of the F and BS states, E_F and E_{BS}, have been used to evaluate the exchange-coupling constant (J) between the iron and manganese atoms in these complexes, using the expression $J = 2(E_F - E_{BS})/(n_1 \times n_2)$ which is applicable to the $J\ \mathbf{S}_1.\mathbf{S}_2$ convention (n_1 and n_2 are the numbers of unpaired electrons on Fe and Mn). To prevent spurious ligand-field contributions to the J values, the orbital holes in the half-filled-minus-one high-spin d^4 configurations of the Mn^{3+} ions in the F and BS solutions were inspected to verify that they were the same. In all three complexes with an Mn^{3+} ion, the listed structures produced in both spin states a hole orbital with the contour of a slightly admixed dz^2 orbital with the z-axis directed along the Mn–O_{bridge} bond. The resulting J values are given in Table S11 and show that protonation of the oxido bridge reduces the value of J like it does in diiron complexes. Table S11 also gives selected metric data which show the expected changes depending on the protonation state of the ligand bridge and the oxidation state of the manganese ion: the hydroxido bridge has longer bond distances than the oxido bridge and the metal–ligand distances of Mn^{4+} are shorter those than of Mn^{3+}. The shorter bonds of the oxido-bridged species compared to

those in the hydroxido-bridged complex result in larger *J* values. Since *J* for the oxido-bridged species is larger than for the hydroxido-bridged complex, its spin related energetics has a larger impact on the structure, causing the bond-length differentials between the BS and F structures for the oxido-bridged species to be larger than those for the hydroxido-bridged complex. The smaller Fe–O_{bridge}–Mn bond angle for the F states (Table S11) are consistent with the slightly longer Mn–O_{bridge} and Fe–O_{bridge} distances for this state compared to the BS state. (N.B. Shorter bridging distances lower the energy of the antiferromagnetic state.) Using the $Fe^{3+}OMn^{3+}$ complex as an example (Table S11), the bond-length differentials between the two spin states (Table S11) correspond with a change of about 4° in the bond angle (compared to 3° from DFT optimized structures, *cf.* Table S11), under the assumption that the Fe···Mn distance has been kept rigorously constant by the two $[poat]^{3-}$ ligand arms that coordinate both Mn and Fe. The A^{SD} values were evaluated with the properties module of Gaussian 09.

Crystallography.

[(TMTACN)Fe^{III}–(μ-O)–Mn^{III}poat]OTf. A purple crystal of approximate dimensions 0.078 x 0.232 x 0.385 mm was mounted in a cryoloop and transferred to a Bruker SMART APEX II diffractometer system. The APEX2[10] program package was used to determine the unit-cell parameters and for data collection (120 sec/frame scan time). The raw frame data was processed using SAINT[11] and SADABS[12] to yield the reflection data file. Subsequent calculations were carried out using the SHELXTL[13] program package. There were no systematic absences nor any diffraction symmetry other than the Friedel condition. The centrosymmetric triclinic space group $P\bar{1}$ was assigned and later determined to be correct.

The structure was solved by direct methods and refined on F^2 by full-matrix least-squares techniques. The analytical scattering factors[14] for neutral atoms were used throughout the analysis. Hydrogen atoms were included using a riding model. There were 1.5 molecules of dichloromethane solvent present. One molecule of dichloromethane was disordered in a general position and one-half molecule of dichloromethane was located on a site also occupied by a half-molecule of triflate. A

second half-molecule of triflate was disordered about an inversion center. Several restraints were employed during the refinement.

Least-squares analysis yielded wR2 = 0.2047 and Goof = 1.046 for 722 variables refined against 13939 data (0.78 Å), R1 = 0.0731 for those 10397 data with I > 2.0σ(I).

There were several high residuals present in the final difference-Fourier map. It was not possible to determine the nature of the residuals. The SQUEEZE[15] routine in the PLATON[16] program package was used to account for the electrons in the solvent accessible voids.

[(TMTACN)Fe^{III}–(μ-O)–Mn^{III}Hpoat](OTf)$_2$. A green crystal of approximate dimensions 0.206 x 0.279 x 0.280 mm was mounted on a glass fiber and transferred to a Bruker SMART APEX II diffractometer system. The APEX2[10] program package was used to determine the unit-cell parameters and for data collection (30 sec/frame scan time). The raw frame data was processed using SAINT[11] and SADABS[12] to yield the reflection data file. Subsequent calculations were carried out using the SHELXTL[13] program package. The diffraction symmetry was 2/*m* and the systematic absences were consistent with the monoclinic space group $P2_1/c$ that was later determined to be correct.

The structure was solved by direct methods and refined on F^2 by full-matrix least-squares techniques. The analytical scattering factors[14] for neutral atoms were used throughout the analysis. Hydrogen atom H(2) was located from a difference-Fourier map and refined (x,y,z and U_{iso}). The remaining hydrogen atoms were included using a riding model. There was one molecule of chloroform solvent present.

Least-squares analysis yielded wR2 = 0.0914 and Goof = 1.034 for 791 variables refined against 13112 data (0.81 Å), R1 = 0.0344 for those 10852 data with I > 2.0σ(I).

There were several high residuals present in the final difference-Fourier map. It was not possible to determine the nature of the residuals although it was probable that *n*-hexane solvent was

present. The SQUEEZE[15] routine in the PLATON[16] program package was used to account for the electrons in the solvent accessible voids.

[(TMTACN)Fe^{II}–(μ-OH)–Mn^{III}poat]OTf. A green crystal of approximate dimensions 0.098 x 0.157 x 0.160 mm was mounted in a cryoloop and transferred to a Bruker SMART APEX II diffractometer system. The APEX2[10] program package was used to determine the unit-cell parameters and for data collection (120 sec/frame scan time). The raw frame data was processed using SAINT[11] and SADABS[12] to yield the reflection data file. Subsequent calculations were carried out using the SHELXTL[13] program package. There were no systematic absences nor any diffraction symmetry other than the Friedel condition. The centrosymmetric triclinic space group $P\bar{1}$ was assigned and later determined to be correct.

The structure was solved by direct methods and refined on F^2 by full-matrix least-squares techniques. The analytical scattering factors[14] for neutral atoms were used throughout the analysis. Hydrogen atoms H(1) and H(8) were located from a difference-Fourier map and refined (x,y,z and U_{iso}). The remaining hydrogen atoms were included using a riding model. There were two molecules of the formula-unit present (Z = 4). Several atoms were disordered and included using multiple components with partial site-occupancy-factors.

Least-squares analysis yielded wR2 = 0.0978 and Goof = 1.001 for 1369 variables refined against 24005 data (0.78 Å), R1 = 0.0474 for those 16338 data with $I > 2.0\sigma(I)$.

Note on the preparative work: The analogous MnFe series, [(TMTACN)Mn-(O(H))-Fe(H)poat]^n, has been prepared and fully characterized and the complexes have distinct properties from those reported here – these findings will be published in a separate report (and can be found in the dissertation: *Synthetic Models for Metalloenzyme Active Sites: Accessing High-Valent Bimetallic Complexes with [M–(μ-O)–M'] Cores* by J.L. Lee 2021 at proquest.com). These comparisons indicate that the metal ions do not scramble during preparation.

Preparation of K[Mn^{II}poat]. To a solution of H_3poat (0.200 g, 0.268 mmol) in anhydrous THF (5 mL) was added KH (0.0340 g, 0.848 mmol), and the reaction was allowed to proceed until gas evolution ceased and all solids were dissolved. To the solution was added $Mn^{II}(OAc)_2$ (0.0520 g, 0.301 mmol). The mixture was stirred for 3 hours and then filtered through a fine fritted glass funnel to remove insoluble material. All volatiles in the pale-yellow filtrate were removed under vacuum to afford an off-white paste. The residues were re-dissolved in 3 mL THF, and 20 mL Et_2O was added to precipitate the product. The mixture was allowed to triturate for 20 min, and the white solids were then collected and dried on a fine fritted glass funnel (70 – 80 % yield). Single crystals were obtained by layering a concentrated K[Mn^{II}poat] solution in CH_2Cl_2 under pentane in low yield (< 10 %). Elemental analysis calc. (found) for K[Mn^{II}poat]·0.5C_5H_{12}, $C_{42}H_{42}KMnN_4O_3P_3\cdot 0.5C_5H_{12}$; C, 61.16 (61.39); H, 5.54 (5,43); N, 6.41 (6.72) %. X-band, ⊥-mode EPR (CH_2Cl_2:THF) g = 7.81, 4.54, 2.85, 1.67, 1.32.

Preparation of [(TMTACN)Fe^{III}–(μ-O)–Mn^{III}poat]OTf. K[Mn^{II}poat] (0.0590 g, 0.0700 mmol) and 18-crown-6 (0.0400 g, 0.151 mmol) were dissolved in 2 mL anhydrous DMF, and mixed for 10 min. [Fe^{II}(TMTACN)(OTf)$_2$] (0.0372 g, 0.0708 mmol) was added in one portion, and the pale solution turned yellow immediately, and was allowed to stir for an additional 15 min. The reaction vial was placed in a pre-cooled aluminum block and chilled in a –35 °C freezer for 20 minutes. IBX-iPr (0.0314 g, 0.0972 mmol) was then added in one portion to the mixture, which turned dark brown. The reaction was allowed to stir for 5 min inside the Al block, before returning to chill inside the freezer for 5 min; this is repeated for eight times (a total of 40 min of stirring time). The solution was then warmed up to room temperature, and all volatiles were removed under vacuum. The residue was redissolved in CH_2Cl_2 and was layered with diethyl ether to yield a brown powder over three days. After the brown powder was collected and dried, it was redissolved in CH_2Cl_2, and was layered with pentane to yield dark brown crystalline needles. The crystals were collected on a glass frit and dried under vacuum,

affording the product in yields that ranged from 30 – 40 %. Elemental analysis calcd. (found) for [(TMTACN)Fe^{III}–(μ-O)–Mn^{III}poat]OTf·1.5CH_2Cl_2, $C_{52}H_{63}F_3FeMnN_7O_7P_3S$·1.5$CH_2Cl_2$; C, 48.74 (48.90); H, 5.05 (5.20); N, 7.44 (7.64) %. UV-Vis (CH_2Cl_2 solution λ_{max}/nm (ε_{max}/$M^{-1}cm^{-1}$)) 348 (sh), 400 (sh), 440 (sh), 490 (sh), 570 (sh), 795 (560). FTIR (ATR, cm^{-1}): 3061, 3052, 3008, 2954, 2903, 2884, 2857, 2825, 1588, 1499, 1464, 1436, 1350, 1259, 1223, 1192, 1153, 1111, 1078, 1066, 1056, 1031, 1011, 991, 961, 919, 896, 836, 801, 748, 720, 695, 636. EPR (X-band, ⊥-mode, CH_2Cl_2:THF, 17.4 K): $g_{x,y,z}$ = 2.042, 2.031, 2.007; $A_{x,y,z}$ = 215, 243, 341 MHz. Mössbauer (5 mM ^{57}Fe-enriched sample, PrCN, 4.2 K) δ = 0.53 mm/s; ΔE_Q = –1.84 mm/s. $E_{1/2}$ (CH_2Cl_2, V versus $[FeCp_2]^{+/0}$): +0.20.

Synthesis of K[Mn^{III}poat(OH)]. To a solution of H_3poat (0.400 g, 0.536 mmol) in anhydrous THF (14 mL) was added KH (0.0665 g, 1.66 mmol), and the reaction was allowed to proceed until gas evolution ceased and all solids were dissolved. To the solution was added $Mn^{II}(OAc)_2$ (0.0980 g, 0.566 mmol). The pale-yellow solution was stirred for 3 hours, then H_2O (10 μL, 0.55 mmol) was added via syringe. After 15 minutes, the reaction mixture was filtered through a medium porosity glass frit to remove insoluble material. KH (0.0225 g, 0.561 mmol) was added to the filtrate, which was allowed to stir for 1 hour. The reaction mixture was then filtered through a medium porosity glass frit to remove any insoluble material. 0.5 equivalent of I_2 (0.0681 g, 0.268 mmol) was added to the pale-yellow solution, which turned dark green and stirred for 30 minutes. The reaction mixture was filtered using a fine porosity glass frit and then concentrated until ~ 3 mL THF remained. Excess pentane (30 mL) was added to crash out the product, which was triturated with pentane until the residue was a free-flowing powder. The green powder was collected on a fine porosity glass funnel, washed with 2 portions of 20 mL pentane, and dried (80 – 90 %). Elemental analysis calc. (found) for K[Mn^{III}poat(OH)]·0.5KI·0.5H_2O, $C_{42}H_{43}KMnN_4O_4P_3$·0.5KI·0.5H_2O; C, 53.28 (53.17); H, 4.68 (4.53); N, 5.92 (5.98) %. FTIR (diamond ATR, cm^{-1}): 3197 (br, OH), 3071, 3045, 3018, 3007, 2076, 2953,

2900, 2855, 2837, 1589, 1572, 1481, 1434, 1307, 1266, 1234, 1165, 1112, 1067, 1025, 996, 963, 927, 860, 794, 746, 717, 694.

Preparation of [(TMTACN)Fe^{II}–(μ-OH)–Mn^{III}poat]OTf. K[Mn^{III}poat(OH)] (0.0879 mg, 0.103 mmol) was dissolved in 4 mL anhydrous CH_2Cl_2. NMe_4OAc (0.0212 mg, 0.111 mmol) was added in one portion, and the mixture was allowed to stir for one hour. The reaction mixture was filtered with a fine porosity glass frit, and the filtrate was added dropwise to a 1 mL CH_2Cl_2 solution of [Fe^{II}(TMTACN)$(OTf)_2$] (0.0540 mg, 0.103 mmol). The reaction was allowed to proceed for one hour, and the mixture was filtered with a medium porosity glass frit to remove any insoluble materials. The filtrate was layered with Et_2O to yield a green powder. After the green powder was collected and dried, it was redissolved in CH_2Cl_2, and was layered with hexane to yield green crystals. The crystals were collected on a glass frit and dried under vacuum, affording the product in yields that ranged from 20-30 %. UV-vis λ_{max}(CH_2Cl_2)/nm (ε/$M^{-1}cm^{-1}$): 470 (sh), 705 (460). $E_{1/2}$ (CH_2Cl_2, V versus $[FeCp_2]^{+/0}$): –1.20, –0.38. Multiple attempts at elemental analysis were unsuccessful. Crystallographic information is found in Figure S7, Tables S1 and S2.

Synthesis of [(TMTACN)Fe^{III}–(μ-O)–Mn^{III}Hpoat]$(OTf)_2$. *Method 1*: [(TMTACN)Fe^{II}–(μ-OH)–Mn^{III}poat]OTf (0.0401 g, 0.0336 mmol) was dissolved in 2 mL anhydrous CH_2Cl_2, and solid [$FeCp_2$]OTf (0.0119 g, 0.0355 mmol) was added in one portion to the green solution. The solution immediately turned dark brown and was allowed to stir for 1.5 hours. All volatiles were then removed under vacuum. The residues were triturated with 20 mL pentane, which was then removed by decanting; the washing process was repeated three times. The residues were redissolved in 3 mL CH_2Cl_2, which was layered with pentane to yield green crystalline needles (70 – 80 %). Single crystals suitable for X-ray diffraction were obtained from layering a concentrated FeMn solution in $CHCl_3$ under hexane.

Method 2: [(TMTACN)Fe^{III}–(μ-O)–Mn^{III}poat]OTf (0.0223 g, 0.0187 mmol) was dissolved in 2 mL anhydrous CH_2Cl_2, and solid 2,6-lutidinium triflate (0.0048 g, 0.0187 mmol) was added in one portion to the brown solution. The solution immediately turned greenish brown and was allowed to stir for 1

hour. All volatiles were then removed under vacuum. The residues were triturated with 20 mL Et_2O, which was then removed by decanting; the washing process was repeated with 20 mL pentane, and a second portion of 20 mL Et_2O. The residues were dried under vacuum then redissolved in 3 mL CH_2Cl_2, which was layered under pentane to yield green crystals (80 – 90 %).

FTIR (diamond ATR, cm^{-1}): 3466, 3260 (NH), 3070, 3057, 2977, 2905, 2869, 2824, 1591, 1550, 1464, 1438, 1363, 1254, 1223, 1146, 1119, 1070, 1028, 1009, 990, 969, 919, 896, 854, 812, 788, 749, 725, 696. EPR (X-band, ⊥-mode, CH_2Cl_2:THF, 17.4 K): $g_{x,y,z}$ = 2.026, 2.024, 2.007; $A_{x,y,z}$ = 209, 255, 323 MHz. UV-vis λ_{max}(CH_2Cl_2)/nm (ε/$M^{-1}cm^{-1}$): 394 (sh), 426 (sh), 525 (sh), 570 (520), 648 (510). Multiple attempts at elemental analysis were unsuccessful.

Low-temperature UV-vis solution studies. In a typical experiment, a 10 mM solution of a FeMn compound was prepared in CH_2Cl_2 at room temperature and kept in a –35 °C freezer for the duration of the experiment. A 30 – 60 μL aliquot of stock metal complex was added via air-tight syringe to the solvent mixture (3 mL) in a 1-cm quartz cuvette to give the desired concentration for oxidation experiments (0.10 – 0.20 mM). The cuvette was then sealed with a rubber septum and precooled to the desired temperature in an 8453 Agilent UV-vis spectrophotometer equipped with a Unisoku Unispeks cryostat. The solution of metal complex was allowed to equilibrate to the desired temperature for at least 15 min. Stock solutions of other reagents were prepared at concentrations between 10 and 100 mM in the same solvent and added via gas-tight syringe. Reactions were monitored spectrophotometrically by UV-vis spectroscopy.

Preparation of EPR solution samples at low temperature. In a typical experiment, a 5 – 6 mM solution of the MnFe compound was prepared in CH_2Cl_2 at room temperature and kept in a –35 °C freezer of the glovebox for the duration of the experiment. A 200 μL aliquot of stock metal complex was added via air-tight syringe to an EPR tube, which was then sealed with a rubber septum and precooled to the desired temperature in a cold bath outside the glovebox. The solution of metal

complex was allowed to equilibrate to the desired temperature for at least 15 min. Stock solutions of other reagents were prepared at concentrations between 40 and 250 mM in the same solvent and added via gas-tight syringe. The typical target final concentration is 5 mM.

Preparation of Mössbauer solution samples at room-temperature. The Mössbauer sample for $[^{57}Fe^{III}(O)Mn^{III}poat]^{+}$ was prepared at room temperature. A 2.75 mM solution of the 96% ^{57}Fe-enriched $[^{57}Fe^{III}(O)Mn^{III}poat]OTf$ (0.0055 g, 0.0046 mmol) was prepared in PrCN, and an aliquot of 0.6 mL was transferred into a Mössbauer cup before freezing in liquid nitrogen.

Preparation of Mössbauer solution samples at low-temperature. In a typical experiment to generate $[^{57}Fe^{III}(O)Mn^{IV}poat]^{2+}$, a 2.75 mM solution of the 96% ^{57}Fe-enriched $[^{57}Fe^{III}(O)Mn^{III}poat]OTf$ (0.0055 g, 0.0046 mmol, 1.12 mL) was prepared in PrCN at room temperature in a glovebox under argon atmosphere, and kept in a –35 °C freezer for the duration of the experiment. A 1.0 mL aliquot of stock metal complex (0.0027 mmol) was added via syringe to a 1-cm quartz. The cuvette was then sealed with a rubber septum and precooled to –80 ºC in a Unisoku Unispeks cryostat typically used for UV-vis experiments outside the glovebox. The solution of metal complex was allowed to equilibrate to the desired temperature for at least 20 min. A stock solution of $[N(p\text{-}C_6H_4Me)_3]OTf$ was prepared at 41.25 mM in PrCN, and an aliquot of the oxidant (0.0041 mmol, 0.10 mL) was added via a gas-tight syringe. Upon completion of the reaction, the septum was sliced open with a razor under a strong flow of argon. The rubber septum was removed and the content in the cuvette was quickly poured into liquid nitrogen. The frozen solid was ground into fine powder and then packed into a pre-cooled Mössbauer cup.

GC-MS experiments. Typically, the solution from a UV-vis substrate reaction experiment with 4-R-PhOH is directly subjected to a GC-MS measurement without further workup (Figure S6). The percent conversion from 4-R-PhOH to bisphenol is calculated by comparing the relative area of

bisphenol with unreacted 4-R-PhOH. Additionally, the following factors are taken into account: 1) 4-R-PhOH was added in 5-fold excess and 2) two equivalents of $[Fe^{III}(O)Mn^{IV}poat]^{2+}$ reacts with two equivalents of 4-R-PhOH to form one equivalent of bisphenol. The percent conversions are calculated to be > 95%.

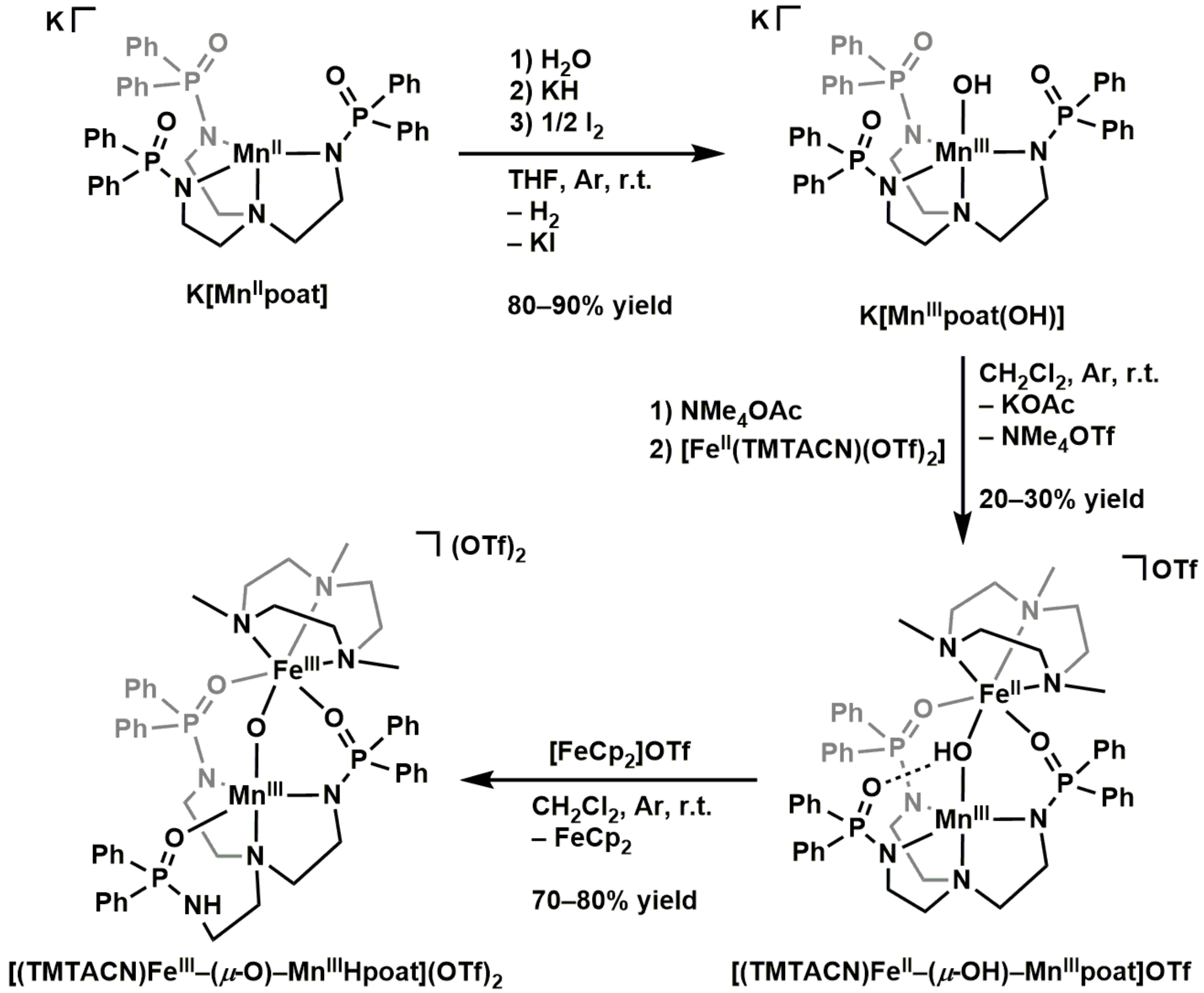

Scheme S1. Preparative routes for $K[Mn^{III}poat(OH)]$, $[Fe^{II}(OH)Mn^{III}poat]OTf$, and $[Fe^{III}(O)Mn^{III}Hpoat](OTf)_2$.

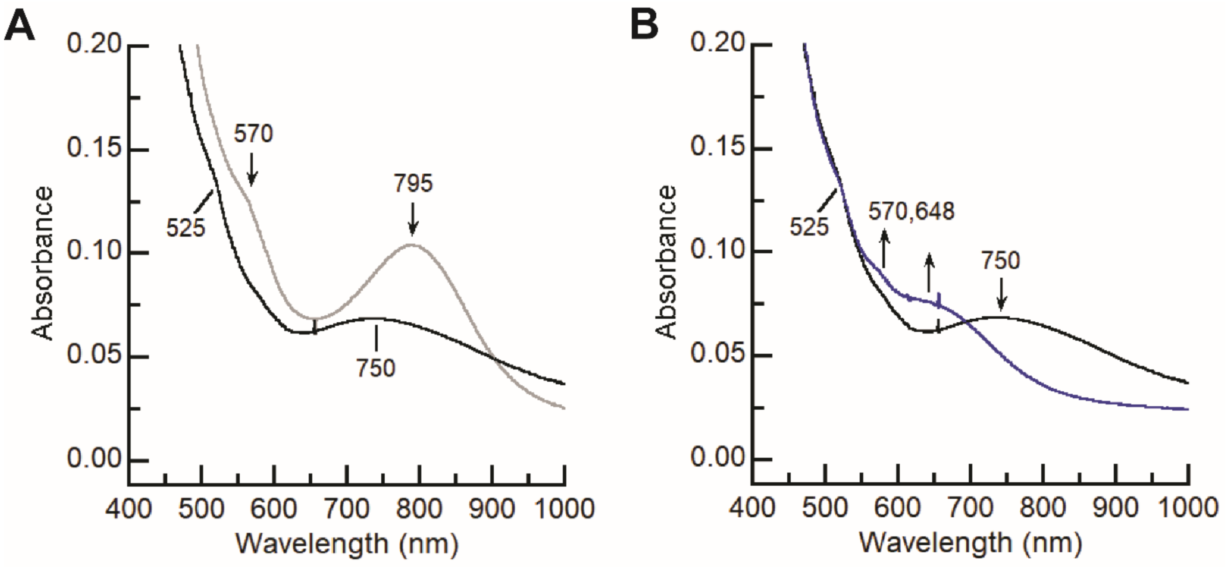

Figure S1. Electronic absorbance spectra of (A) the protonation of [Fe^{III}(O)Mn^{III}poat]$^{+}$ (grey) to generate [Fe^{III}(OH)Mn^{III}poat]$^{2+}$ (black) at –60 °C in CH_2Cl_2 and (B) subsequent conversion to [Fe^{III}(O)Mn^{III}Hpoat]$^{2+}$ (blue).

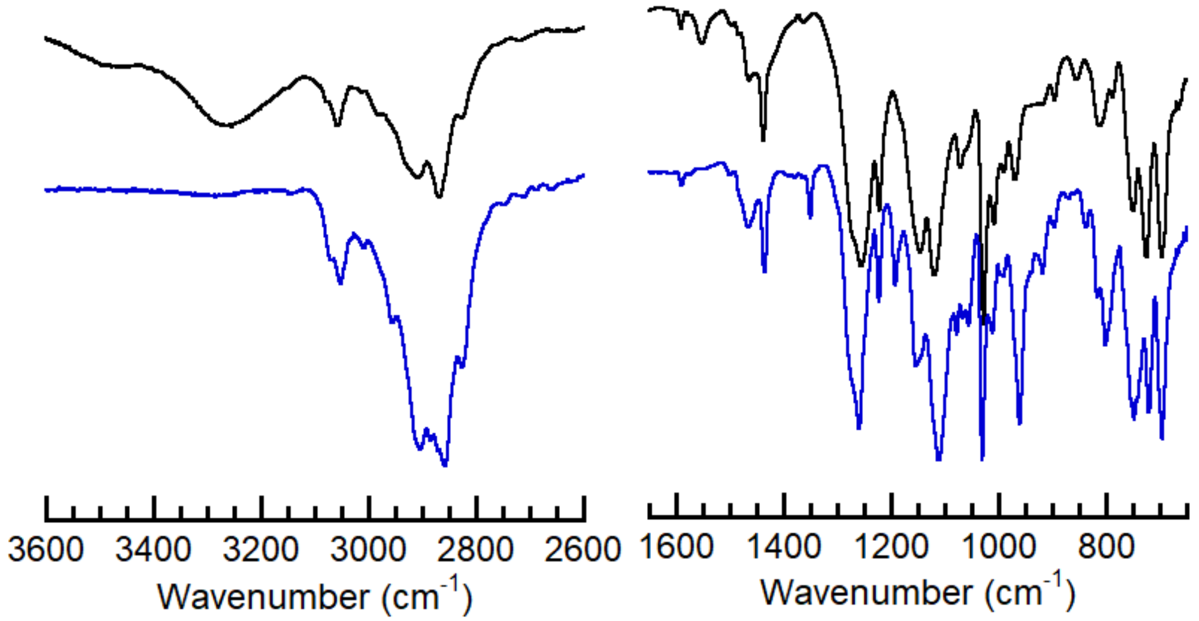

Figure S2. FT-IR spectra of [Fe^{III}(O)Mn^{III}poat]$^{+}$ (blue) and [Fe^{III}(O)Mn^{III}Hpoat]$^{2+}$ (black). The left panel shows the high energy region, and the right panel shows the low energy region.

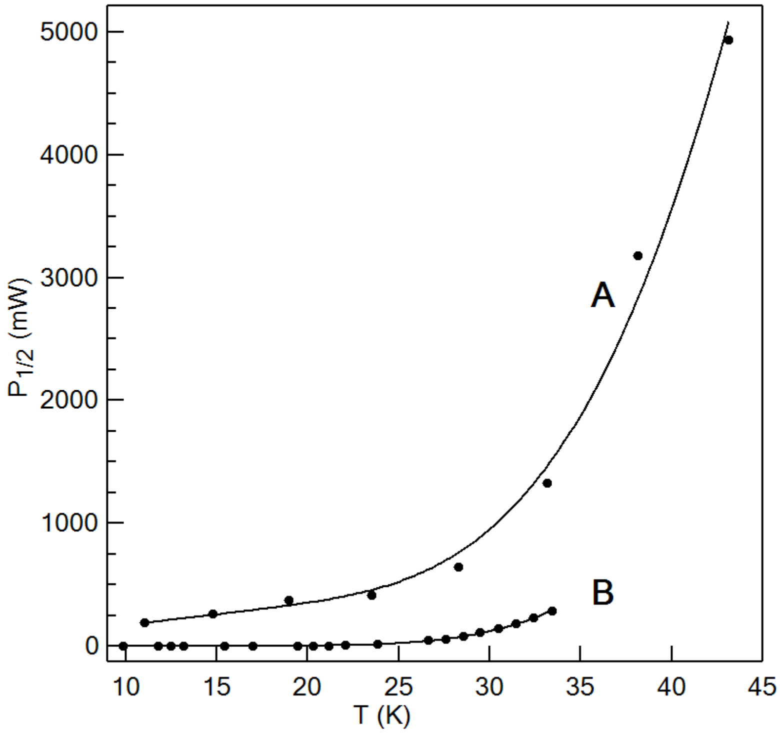

Figure S3. The microwave power required to half-saturate the EPR signal of $[Fe^{III}(O)Mn^{III}Hpoat]^{2+}$ (A) and $[Fe^{III}(O)Mn^{III}poat]^{+}$ (B) versus temperature (dots). The traces are a fit to $P_{1/2} = AT + B/(\exp(\Delta/kT)-1)$ for: (A) A = 17 mW/K, B = 8.1×10^5 mW, and Δ = 150 cm^{-1} and (B) A = 2.2×10^{-2} mW/K, B = 6.7×10^5 mW, and Δ = 180 cm^{-1}. The energy splitting between the ground S = 1/2 and S = 3/2 spin manifolds is Δ = 1.5 J.

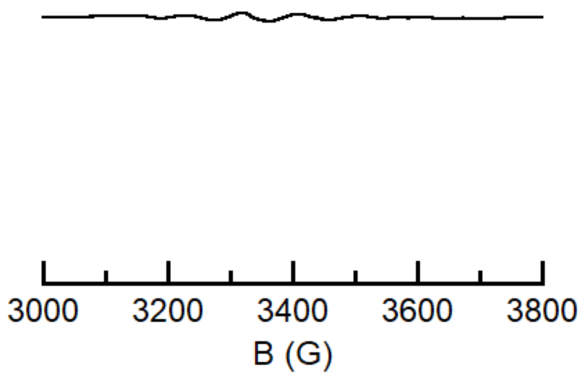

Figure S4. The silent ⊥-mode X-band EPR spectrum of $[Fe^{III}(O)Mn^{IV}poat]^{2+}$ was collected in a 5 mM frozen CH_2Cl_2 solution at 16 K. The spectrum contains ~ 10 % unreacted $[Fe^{III}(O)Mn^{III}poat]^{+}$.

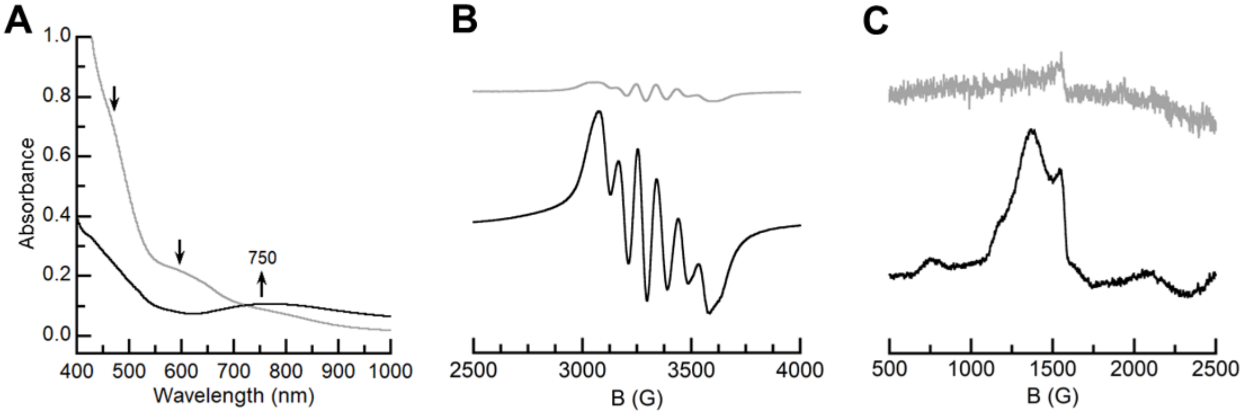

Figure S5. Electronic absorbance (A) and EPR (B-high field, C-low field) spectra of the reaction of $[Fe^{III}(O)Mn^{IV}poat]^{2+}$ towards 4-MeO-PhOH. The spectra show before (grey) and after (black) the addition of 5 equiv. 4-MeO-PhOH to $[Fe^{III}(O)Mn^{IV}poat]^{2+}$ to produce $[Fe^{III}(OH)Mn^{III}poat]^{2+}$ (0.20 mM (UV-vis) and 5.0 mM (EPR) CH_2Cl_2 solution at –60 °C). EPR spectra were collected at 77 K.

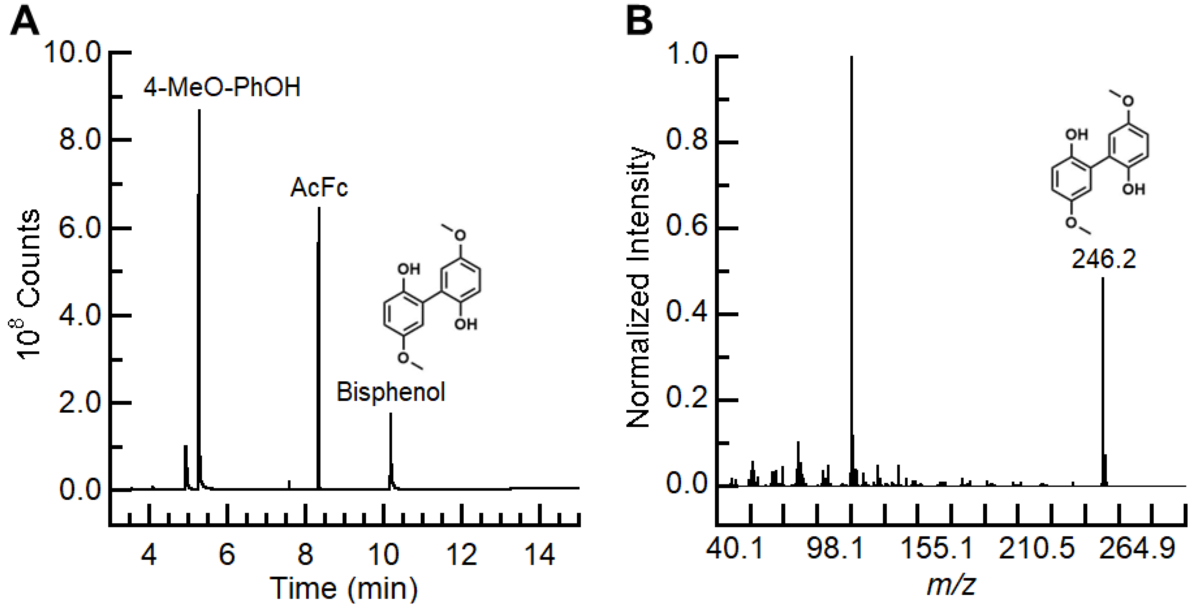

Figure S6. GC-MS plot (A) of the reaction of $[Fe^{III}(O)Mn^{IV}poat]^{2+}$ towards 4-MeO-PhOH. AcFc = $FeCp(C_5H_4C(O)Me)$ byproduct. (B) Mass spectrum of the species detected t = 10.2 min in gas chromatography corresponds to the bisphenol (246.2 *m*/*z*).

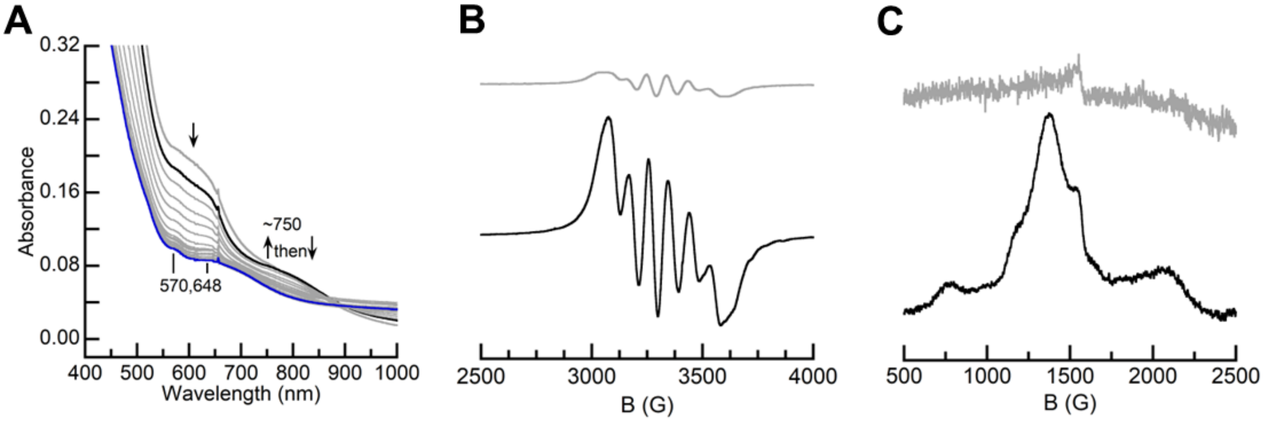

Figure S7. Electronic absorbance (A) and EPR (B-high field, C-low field) spectra of the reaction of $[Fe^{III}(O)Mn^{IV}poat]^{2+}$ (grey) towards 4-^{t}Bu-PhOH. In the UV-vis spectra (0.20 mM CH_2Cl_2 solution at –60 ^{o}C), a feature at ~750 nm suggests the initial generation of $[Fe^{III}(OH)Mn^{III}poat]^{2+}$ (black, 40 sec after the addition of 5 equiv. 4-^{t}Bu-PhOH); however, $[Fe^{III}(OH)Mn^{III}poat]^{2+}$ was unstable with respect to the PCET timescale, and further converted (thin grey traces, 40 sec intervals) to $[Fe^{III}(O)Mn^{III}Hpoat]^{2+}$ (blue). The generation of $[Fe^{III}(OH)Mn^{III}poat]^{2+}$ is demonstrated by the EPR spectra (B, C), where the spectra show before (grey) and after (black) the addition of 5 equiv. 4-MeO-PhOH to $[Fe^{III}(O)Mn^{IV}poat]^{2+}$ to produce $[Fe^{III}(OH)Mn^{III}poat]^{2+}$ (5.0 mM CH_2Cl_2 solution at –60 ^{o}C). EPR spectra were collected at 77 K.

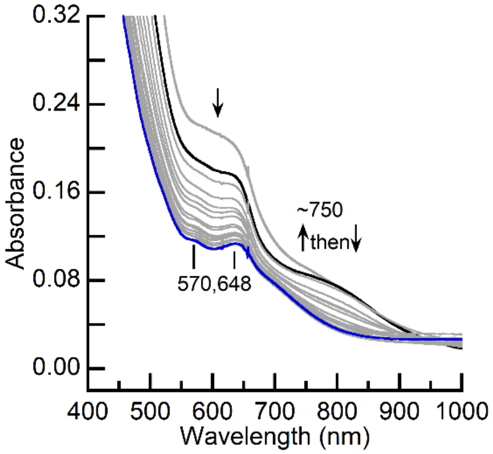

Figure S8. Electronic absorbance spectra of the reaction of $[Fe^{III}(O)Mn^{IV}poat]^{2+}$ (grey) towards 4-F-PhOH. In the UV-vis spectra (0.20 mM CH_2Cl_2 solution at –60 ^{o}C), a feature at ~750 nm suggests the initial generation of $[Fe^{III}(OH)Mn^{III}poat]^{2+}$ (black, 60 sec after the addition of 5 equiv. 4-F-PhOH); however, $[Fe^{III}(OH)Mn^{III}poat]^{2+}$ was unstable with respect to the PCET timescale, and further converted (thin grey traces, 60 sec intervals) to $[Fe^{III}(O)Mn^{III}Hpoat]^{2+}$ (blue).

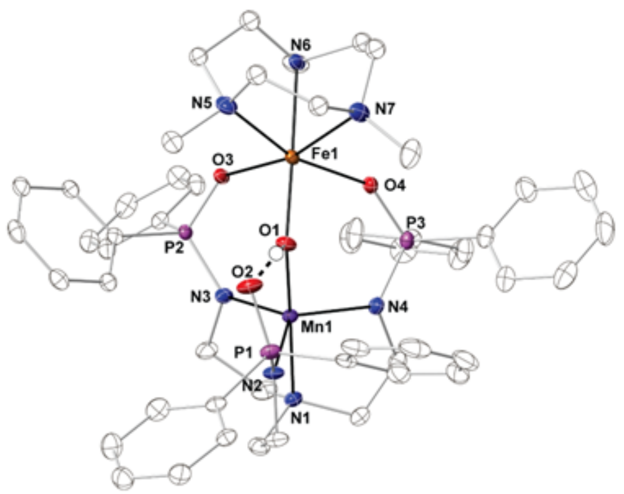

Figure S9. Thermal ellipsoid diagram depicting the molecular structure of $[Fe^{II}(OH)Mn^{III}poat]^{+}$. Ellipsoids are drawn at the 50 % probability level, and only the hydroxido H atom is show for clarity. The triflate counter anion is outer-sphere and is not interacting with the cation. Metrical parameters are shown in Table S1.

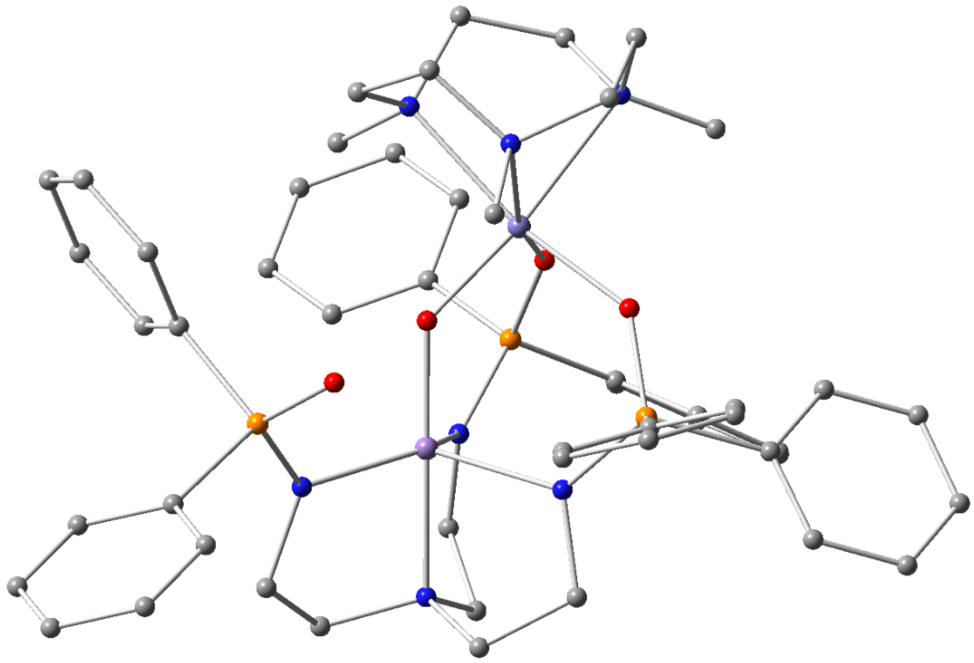

Figure S10. DFT optimized structure of $[(TMTACN)Fe^{III}–(\mu\text{-}O)–Mn^{III}poat]^{+}$ in BS state (Table S3). H atoms are not shown for clarity.

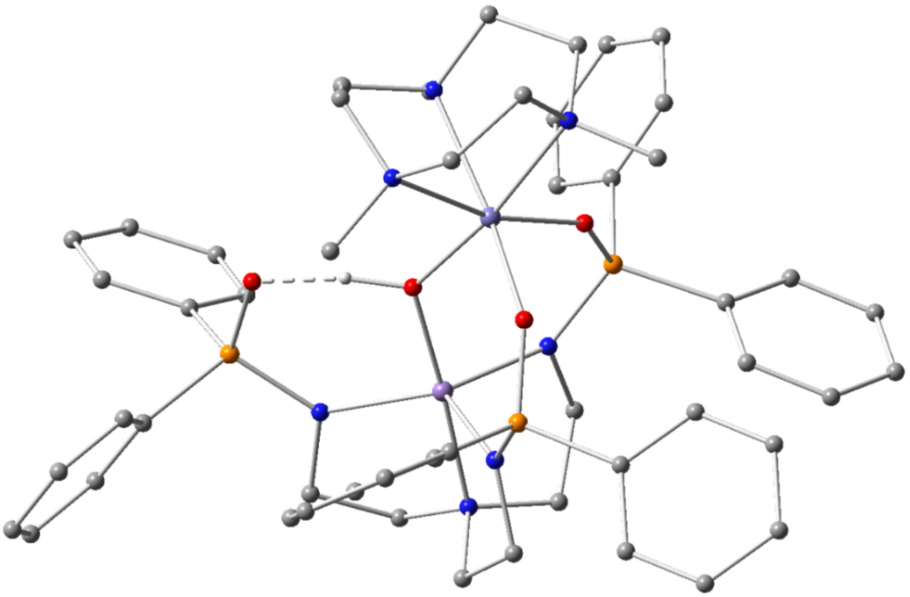

Figure S11. DFT optimized structure of [(TMTACN)Fe^{III}–(*μ*-OH)–Mn^{III}poat]$^{2+}$ in BS state (Table S5). For clarity, only the H atom at the hydroxido bridge is shown. The dashed line is a H-bond of 1.44 Å.

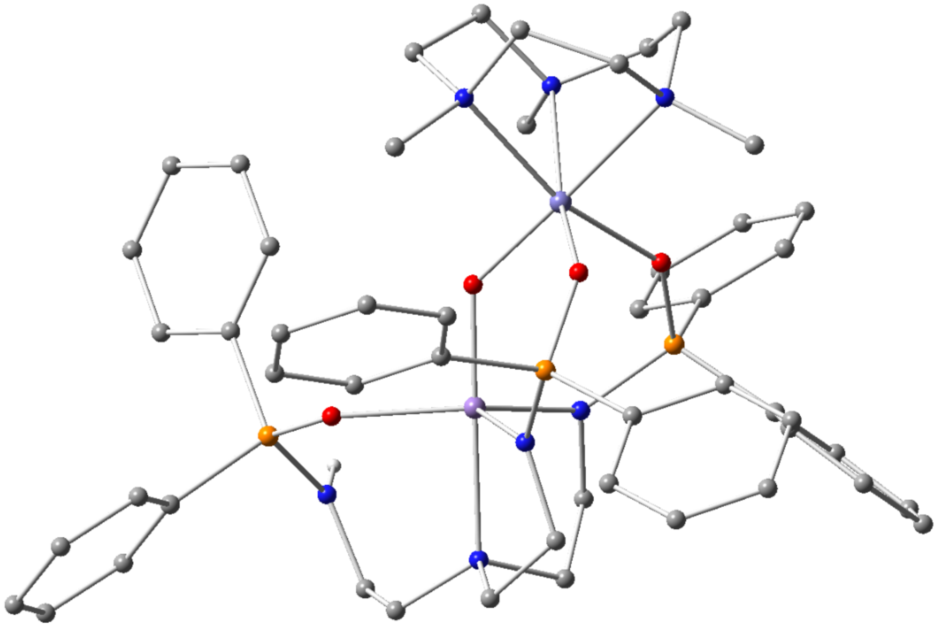

Figure S12. DFT optimized structure of [(TMTACN)Fe^{III}–(*μ*-O)–Mn^{III}Hpoat]$^{2+}$ in BS state (Table S7). For clarity, only the H atom of the phosphinic amide arm in [poat]$^{3-}$ is shown.

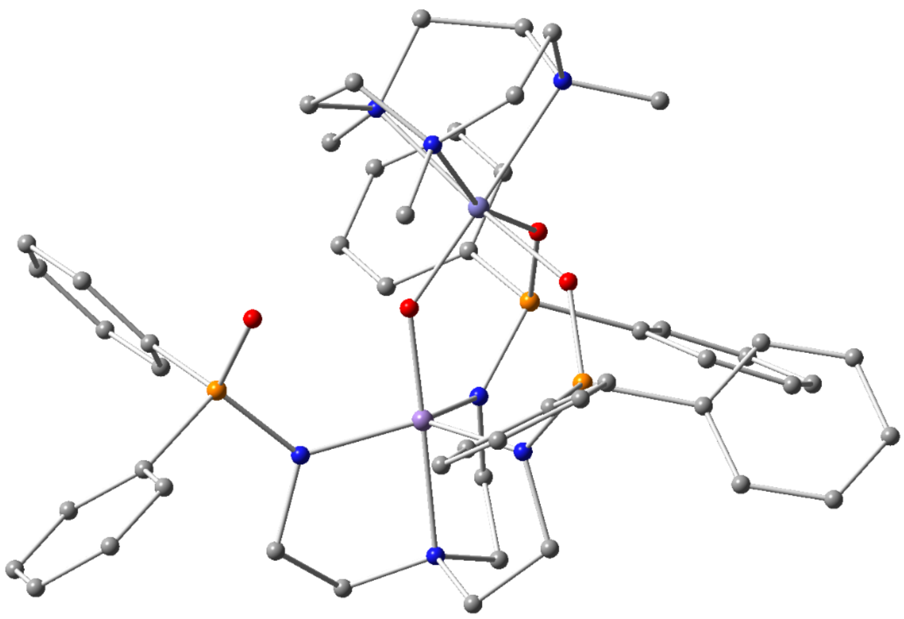

Figure S13. DFT optimized structure of [(TMTACN)Fe^{III}–(μ-O)–Mn^{IV}poat$]^{2+}$ in BS state (Table S9). H atoms are not shown for clarity.

Table S1. Selected bond lengths/distances (Å) and angles (°) for $[Fe^{II}(OH)Mn^{III}poat]^{+a}$.

	$[Fe^{II}(OH)Mn^{III}poat]^{+}$
	Bond Lengths/Distances (Å)
Mn1–N1	2.053(2)
Mn1–N2	2.024(2)
Mn1–O2	–
Mn1–N3	2.061(2)
Mn1–N4	2.065(2)
Mn1–O1	1.845(2)
O1···O2	2.660(3)
Fe1–O1	2.022(2)
Fe1–O3	2.091(2)
Fe1–O4	2.066(2)
Fe1–N5	2.264(2)
Fe1–N6	2.214(2)
Fe1–N7	2.291(2)
Mn1···Fe1	3.433(1)
av Mn1–N/O_{eq}	2.050(2)
av Fe1–N_{TMTACN}	2.256(2)
d[Mn1–N_{eq}]^b	0.295
d[Fe1–N_{TMTACN}]^b	1.533
	Angles (°)
O1–Mn1–N1	177.05(9)
N2–Mn1–N3	124.08(9)
O2–Mn1–N3	–
N3–Mn1–N4	108.05(9)
N2–Mn1–N4	121.67(9)
O2–Mn1–N4	–
Mn1–O1–Fe1	125.10(10)
O3–Fe1–O4	101.37(7)
N5–Fe1–N6	79.53(8)
N5–Fe1–N7	77.67(9)
N6–Fe1–N7	79.34(9)
	Calculated Values
$\tau_5{}^b$	0.883

[a] There are two crystallographically different, but chemically equivalent, molecules in the asymmetric unit, and the bond lengths, distances, and angles are reported as an average.
[b] d[M–N_x] denotes the displacement of the metal atom from the 3-nitrogen-atom plane. N_{eq} represents the plane formed by N2,N3,N4, and N_{TMTACN} represents the plane formed by N5,N6.N7.
[c] Trigonality structural parameter, $\tau_5 = (\beta - a)/60°$. β is the largest bond angle observed, and a is the second largest bond angle observed.

Table S2. Crystallographic data for $[Fe^{III}(O)Mn^{III}poat]^{+}$, $[Fe^{III}(O)Mn^{III}Hpoat]^{2+}$, and $[Fe^{II}(OH)Mn^{III}poat]^{+}$.

	[(TMTACN)Fe^{III}–(*μ*-O)–Mn^{III}poat]OTf	[(TMTACN)Fe^{III}–(*μ*-O)–Mn^{III}Hpoat]$(OTf)_2$	[(TMTACN)Fe^{II}–(*μ*-OH)–Mn^{III}poat]OTf
Formula	C_{52} H_{63} F_3 Fe Mn N_7 O_7 P_3 S·1.5(CH_2Cl_2)	C_{53} H_{64} F_6 Fe Mn N_7 O_{10} P_3 S_2·$CHCl_3$	C_{52} H_{64} F_3 Fe Mn N_7 O_7 P_3 S
fw	1318.24	1460.30	1191.86
T (K)	133(2)	93(2)	93(2)
Crystal system	Triclinic	Monoclinic	Triclinic
Space group	$P\bar{1}$	$P2_1/c$	$P\bar{1}$
a (Å)	14.5521(14)	16.7414(9)	14.8784(8)
b (Å)	15.0662(15)	14.8414(8)	17.2401(9)
c (Å)	16.7358(16)	26.8159(15)	21.4396(11)
α (°)	70.9679(15)	90	90.8210(10)
β (°)	65.6681(15)	92.1641(10)	90.3550(9)
γ (°)	82.9150(16)	90	98.1446(9)
Z	2	4	4
V ($Å^3$)	3160.3(5)	6658.1(6)	5443.2(5)
δ_{calc} (mg/m^3)	1.385	1.457	1.454
Independent reflections	13939	13112	24005
R1	0.0731	0.0344	0.0474
wR2	0.2047	0.0914	0.0978
Goof	1.046	1.034	1.001
CCDC#	2287843	2287844	2287842

$wR2 = [\Sigma[w(F_o^2-F_c^2)^2] / \Sigma[w(F_o^2)^2]]^{1/2}$

$R1 = \Sigma||F_o|-|F_c|| / \Sigma|F_o|$

Goof = $S = [\Sigma[w(F_o^2-F_c^2)^2] / (n-p)]^{1/2}$ where n is the number of reflections and p is the total number of parameters refined.

The thermal ellipsoid plots are shown at the 50% probability level.

Table S3. [(TMTACN)Fe^{III}–(μ-O)–Mn^{III}poat]$^{+}$, BS (5/2, –2) optimized.

Fe	0.77929639	0.30877229	1.71238766
P	0.94978655	-2.65382016	0.11642371
P	2.26317894	1.78379209	-0.95894751
P	-3.64999638	0.07226906	-0.36129887
O	-0.50045557	0.46398937	0.44440132
O	1.28604219	-1.60652010	1.23891622
O	2.17949637	1.24239958	0.50884986
O	-3.33269662	-0.99201472	0.69029533
N	-0.59719141	-0.46894083	-3.32723960
N	0.26993575	-1.93535125	-1.18766332
N	1.06139509	1.20322815	-1.90976648
N	-2.53603048	0.22743718	-1.60444744
N	2.40622016	0.46633389	3.35083193
N	0.19225324	2.24035068	2.77462460
N	-0.31854030	-0.49019126	3.53950219
C	0.14619003	-2.68981815	-2.46023892
C	-0.82892997	-1.95368083	-3.37966743
C	1.18085207	1.26081310	-3.39181296
C	0.70979005	-0.07455720	-3.95972422
C	-2.97889280	0.14469056	-3.02260001
C	-1.74976107	0.30017299	-3.91044733
C	-0.14938284	-3.96655457	0.76208422
C	-1.54017862	-3.83662578	0.59171714
C	-2.40158307	-4.80819914	1.10730039
C	-1.88982539	-5.91490664	1.78999217
C	-0.50908730	-6.04738516	1.96910760
C	0.35801788	-5.07958189	1.45886231
C	2.50941222	-3.53342013	-0.29227880
C	2.50416034	-4.67482310	-1.11655459
C	3.69727259	-5.32528730	-1.43668896
C	4.91220729	-4.85248833	-0.93143450
C	4.92832891	-3.72664968	-0.10427813
C	3.73501502	-3.07082277	0.21195083
C	3.88380313	1.33114658	-1.68148660
C	4.24453503	-0.02916219	-1.66776000
C	5.43968081	-0.45130947	-2.24977934
C	6.28923499	0.47698046	-2.86125824
C	5.93633483	1.82731769	-2.89048354
C	4.73966255	2.25473792	-2.30621373
C	2.25240412	3.61411199	-0.88714970
C	3.22411916	4.31366467	-0.14604948
C	3.18944197	5.70696131	-0.07341206
C	2.18512702	6.41899808	-0.73922573
C	1.21515634	5.73376869	-1.47431130

C	1.24773894	4.33775488	-1.54740927
C	-3.87559945	1.71778881	0.41997985
C	-3.43787973	2.90131732	-0.19280323
C	-3.69245149	4.14376068	0.39780642
C	-4.38713320	4.21425690	1.60836704
C	-4.82237579	3.03881300	2.23095850
C	-4.56884600	1.79834975	1.64154028
C	-5.28784588	-0.27993021	-1.12184256
C	-6.07548892	0.70478397	-1.74304057
C	-7.31231289	0.37412201	-2.30137163
C	-7.77799442	-0.94291949	-2.24382126
C	-7.00763268	-1.92663559	-1.61784811
C	-5.77149209	-1.59747189	-1.05619059
C	-0.98951646	0.63405002	4.28018956
C	0.76175234	-1.14887528	4.34182659
C	1.93302695	-0.20527718	4.61081635
C	2.57327990	1.94563864	3.51018358
C	1.23884704	2.64556629	3.77402291
C	-0.03517724	3.32414245	1.77235023
C	-1.34142309	-1.50491770	3.12398833
C	3.67767527	-0.14705189	2.86856106
C	-1.11705557	1.87283081	3.40464610
H	-0.25376431	-3.70171322	-2.31885713
H	-0.74403161	-2.30622867	-4.41304321
H	-1.84452011	-2.12911373	-3.03717525
H	2.20911068	1.42632719	-3.72661304
H	0.58813282	2.08630327	-3.80012021
H	0.59786787	-0.04119133	-5.04866520
H	1.44031058	-0.83841384	-3.70924121
H	-3.68840197	0.93401034	-3.28759322
H	-3.48920763	-0.79947084	-3.23875451
H	-1.94391492	-0.03283566	-4.93557955
H	-1.44978490	1.34604085	-3.94368231
H	-1.95071993	-2.95868309	0.11230682
H	-3.46929882	-4.68999513	0.98443820
H	-2.56018992	-6.66691932	2.18401768
H	-0.10994616	-6.90121540	2.49981434
H	1.42423917	-5.19559015	1.59678796
H	1.57123759	-5.07489175	-1.48861332
H	3.67738806	-6.20301947	-2.06813080
H	5.83443492	-5.36359703	-1.17190818
H	5.86385988	-3.36536564	0.30151543
H	3.74464375	-2.20681088	0.85720910
H	3.58847957	-0.75372482	-1.20773376
H	5.70330782	-1.49984981	-2.22765670
H	7.21494729	0.14892784	-3.31404895

H	6.58635081	2.54822676	-3.36723610
H	4.47958708	3.30246630	-2.34172149
H	4.00736052	3.77079729	0.36643316
H	3.94274870	6.23654449	0.49403349
H	2.16217150	7.49884035	-0.68580085
H	0.43787625	6.28230864	-1.98880984
H	0.48804063	3.80379231	-2.10027274
H	-2.89208204	2.83869526	-1.12421011
H	-3.35584768	5.05107635	-0.08666327
H	-4.59506238	5.17533795	2.05963338
H	-5.36684591	3.09078209	3.16462965
H	-4.90861442	0.88981470	2.11987919
H	-5.73802232	1.73258237	-1.77249186
H	-7.91403303	1.14149472	-2.76976527
H	-8.73800540	-1.19695477	-2.67257639
H	-7.37268286	-2.94328237	-1.55748300
H	-5.18423815	-2.34637071	-0.54353069
H	-1.98457901	0.31856039	4.59875682
H	1.09909012	-2.00059079	3.75613904
H	2.75306731	-0.77056321	5.06002096
H	1.65698905	0.55544277	5.33886487
H	3.27625117	2.17097542	4.32249408
H	2.99991976	2.30582644	2.57751569
H	0.87997544	2.42641942	4.77784728
H	1.38926927	3.72669516	3.73366706
H	0.91390056	3.62751247	1.34748238
H	-0.51305844	4.18884204	2.24462988
H	-0.67229840	2.94189176	0.98200961
H	-2.04242255	-1.08239697	2.40658593
H	-1.88057331	-1.87389687	4.00347740
H	-0.83638684	-2.33477705	2.64920429
H	3.53572087	-1.21934143	2.77451483
H	4.49740668	0.04913878	3.56914504
H	3.91654823	0.26293770	1.89231428
H	-1.50923591	2.71248688	3.99102904
H	-1.81107943	1.68120644	2.58894424
H	0.36068850	-1.52450006	5.29114262
H	-0.43141379	0.85204819	5.18995632
H	1.11938852	-2.80937733	-2.94962884
Mn	-0.55251528	0.02370427	-1.29314317

Table S4. [(TMTACN)Fe^{III}–(μ-O)–Mn^{III}poat]$^{+}$, F (5/2, 2) optimized.

Fe	0.79643140	0.33080879	1.70066979
P	0.87281539	-2.65379521	0.15278565
P	2.32281842	1.71679443	-0.98435933
P	-3.66643851	0.10278758	-0.39569065
O	-0.52651702	0.53388736	0.47089347
O	1.23319299	-1.59995248	1.26376428
O	2.22159094	1.18816180	0.48837866
O	-3.38581003	-1.02342582	0.60226850
N	-0.60167863	-0.46792508	-3.33251951
N	0.22389002	-1.92652896	-1.16199247
N	1.08750045	1.18899439	-1.92060229
N	-2.54509784	0.28876435	-1.62820368
N	2.41353376	0.46181739	3.34669308
N	0.27539477	2.30210779	2.72480765
N	-0.34011546	-0.39423481	3.53043091
C	0.09651076	-2.69113415	-2.42955610
C	-0.85812070	-1.94893871	-3.36599678
C	1.20456497	1.23238828	-3.40438259
C	0.71429825	-0.10110427	-3.96209632
C	-2.97620651	0.19256379	-3.04985261
C	-1.73811393	0.31425884	-3.93084852
C	-0.26271491	-3.92700645	0.81252606
C	-1.64971255	-3.75380326	0.64956280
C	-2.53747375	-4.69502513	1.17720881
C	-2.05683038	-5.81366143	1.86282836
C	-0.67972247	-5.98852308	2.03465096
C	0.21404887	-5.05127309	1.51373921
C	2.40937816	-3.58439498	-0.23335997
C	2.37433399	-4.73600590	-1.04281300
C	3.54845104	-5.42825614	-1.34487855
C	4.77390500	-4.98824457	-0.83543850
C	4.81946174	-3.85281420	-0.02269569
C	3.64533415	-3.15491602	0.27477363
C	3.91710710	1.18336793	-1.71009275
C	4.22080477	-0.19026720	-1.67514795
C	5.39064492	-0.67283084	-2.26158242
C	6.27097313	0.20777667	-2.89947685
C	5.97460916	1.57106685	-2.94986508
C	4.80391716	2.05902170	-2.36050581
C	2.40610378	3.54553763	-0.92448268
C	3.41722693	4.19915185	-0.19433348
C	3.45457304	5.59282831	-0.13140145
C	2.48416097	6.35090922	-0.79666855
C	1.47604411	5.71143836	-1.52167447

C	1.43612699	4.31510418	-1.58454710
C	-3.85454405	1.71147181	0.46550863
C	-3.40266178	2.91582980	-0.09359651
C	-3.63829516	4.13252913	0.55534180
C	-4.32835652	4.15634022	1.77050095
C	-4.77742825	2.95922393	2.33953191
C	-4.54253174	1.74418411	1.69209382
C	-5.31094469	-0.16554815	-1.17651352
C	-6.04854268	0.86283162	-1.78864476
C	-7.29333860	0.59590974	-2.36285823
C	-7.81702368	-0.70020007	-2.33049817
C	-7.09660357	-1.72669734	-1.71415231
C	-5.85217797	-1.46129424	-1.13683243
C	-0.97647606	0.76652258	4.24570328
C	0.70832500	-1.07780491	4.35415873
C	1.91068138	-0.17230489	4.61544421
C	2.63627665	1.93628598	3.48410411
C	1.32699369	2.68851277	3.72602160
C	0.09528140	3.37356900	1.69952050
C	-1.39526089	-1.37842125	3.12106916
C	3.66483956	-0.20605888	2.88267604
C	-1.05191257	1.99497775	3.34998121
H	-0.32268612	-3.69433003	-2.28265272
H	-0.77179084	-2.31764235	-4.39364819
H	-1.87911429	-2.10268358	-3.02903462
H	2.23354914	1.38194162	-3.74453205
H	0.62134277	2.06251107	-3.81639823
H	0.60801678	-0.07553463	-5.05184423
H	1.43154044	-0.87429630	-3.70158057
H	-3.66997750	0.98969467	-3.33180692
H	-3.50012045	-0.74598074	-3.25768084
H	-1.93252174	-0.02742940	-4.95304751
H	-1.41930772	1.35410795	-3.97504100
H	-2.04104207	-2.86514667	0.17283695
H	-3.60136852	-4.54119269	1.06128323
H	-2.74803229	-6.54188373	2.26560266
H	-0.30419094	-6.85122106	2.56828562
H	1.27678668	-5.19953878	1.64697688
H	1.43215186	-5.11150185	-1.41707127
H	3.50524763	-6.31303500	-1.96520317
H	5.68111476	-5.53185412	-1.06132362
H	5.76289000	-3.51611157	0.38592998
H	3.67832488	-2.28323985	0.90860228
H	3.54049025	-0.87767296	-1.19413272
H	5.61080049	-1.73079359	-2.22170962
H	7.17695004	-0.16692329	-3.35598296

H	6.64886591	2.25520415	-3.44671068
H	4.58787676	3.11613381	-2.41139876
H	4.17530577	3.62020436	0.31655394
H	4.23763752	6.08689110	0.42775356
H	2.51712294	7.43084319	-0.75102826
H	0.72520762	6.29565052	-2.03611244
H	0.64652782	3.81684831	-2.12892547
H	-2.86142616	2.88866001	-1.02925725
H	-3.29172324	5.05688424	0.11173221
H	-4.52290416	5.09833450	2.26591274
H	-5.31933622	2.97500651	3.27604086
H	-4.89403932	0.81890622	2.12788237
H	-5.66551456	1.87496654	-1.79875340
H	-7.85632096	1.39623266	-2.82425989
H	-8.78324461	-0.90475333	-2.77164674
H	-7.50628715	-2.72720110	-1.67389628
H	-5.30160667	-2.24334803	-0.63315808
H	-1.98485577	0.49235405	4.56035449
H	1.01871517	-1.95179326	3.78662172
H	2.70816330	-0.75914551	5.07715197
H	1.65880483	0.60897167	5.33044810
H	3.34198845	2.14703652	4.29770121
H	3.08272951	2.26528589	2.54911219
H	0.95315507	2.49921673	4.73028420
H	1.51588197	3.76271162	3.66828294
H	1.06023176	3.64836082	1.29160288
H	-0.37209526	4.25651485	2.14775864
H	-0.53228934	2.98839017	0.90305676
H	-2.06741627	-0.94775926	2.38217631
H	-1.96208538	-1.70552762	3.99948960
H	-0.91610812	-2.23805166	2.67357562
H	3.48428416	-1.27375957	2.80623199
H	4.48631908	-0.02728842	3.58559152
H	3.92501360	0.17813050	1.90137145
H	-1.41384001	2.85767177	3.92190074
H	-1.74587712	1.81824202	2.53147515
H	0.28588350	-1.42089664	5.30627020
H	-0.41836367	0.97926027	5.15662888
H	1.07161832	-2.83244626	-2.90928584
Mn	-0.56654967	0.04992858	-1.30597828

Table S5. [(TMTACN)Fe^{III}–(μ-OH)–Mn^{III}poat]$^{2+}$, BS (5/2, –2) optimized.

Fe	0.96828097	0.02918452	1.79343367
P	1.13599723	-2.59780620	-0.30646665
P	2.09758899	2.07230995	-0.66864167
P	-3.70472136	0.46494863	-0.18240842
O	-0.56936310	0.22464711	0.64166514
O	1.46181954	-1.69960603	0.95269239
O	2.06131496	1.23512037	0.67129783
O	-2.98492291	0.24912296	1.18200485
N	-0.66192242	0.03045906	-3.30874700
N	0.39024800	-1.70085872	-1.46570078
N	0.86753138	1.59573442	-1.64625083
N	-2.57009614	0.37062948	-1.41429874
N	2.59740698	0.01342972	3.30991870
N	0.25403065	1.70503897	3.17578684
N	-0.04515432	-1.12335435	3.49165222
C	0.38197883	-2.19673168	-2.87243232
C	-0.69580374	-1.44184396	-3.64465145
C	0.88252748	1.96283045	-3.09280332
C	0.55275410	0.71286825	-3.89978939
C	-3.07662653	0.33116099	-2.81690298
C	-1.93382824	0.72234628	-3.74171933
C	0.07952076	-4.00381551	0.17848628
C	-1.27831641	-4.01587116	-0.18487656
C	-2.09876674	-5.08254822	0.19084519
C	-1.57252078	-6.14331145	0.93310742
C	-0.22200661	-6.13965965	1.29997584
C	0.60277131	-5.07927420	0.92262353
C	2.69625419	-3.35899194	-0.86867572
C	2.67801187	-4.48166185	-1.71884848
C	3.87282156	-5.04897298	-2.16452287
C	5.09876345	-4.51321065	-1.75882876
C	5.12722432	-3.40604911	-0.90661313
C	3.93349919	-2.82957796	-0.46525680
C	3.69656647	1.82614988	-1.50808216
C	4.08158949	0.50738343	-1.81928817
C	5.26671670	0.26148059	-2.51113024
C	6.07932408	1.32775633	-2.91265117
C	5.69990621	2.63944489	-2.62251834
C	4.51434718	2.89196741	-1.92567107
C	2.01029408	3.83874974	-0.23098774
C	2.98821654	4.42284938	0.59927395
C	2.90522255	5.77258252	0.94311475
C	1.84816004	6.55512385	0.46372815
C	0.87300266	5.98368143	-0.35680350

C	0.95134015	4.63131417	-0.70190103
C	-4.52392854	2.08907761	-0.25763835
C	-3.87996182	3.17960562	-0.86861929
C	-4.47500942	4.44262703	-0.86468719
C	-5.71488103	4.63063181	-0.24733224
C	-6.35901658	3.55385569	0.36928556
C	-5.77052148	2.28827315	0.36413385
C	-5.00648026	-0.79391191	-0.37949787
C	-6.01699118	-0.67513876	-1.35204238
C	-6.97995110	-1.67620677	-1.49304522
C	-6.95103940	-2.79907390	-0.66087772
C	-5.95806554	-2.91906001	0.31574416
C	-4.98840753	-1.92390845	0.45676634
C	-0.70965475	-0.19197761	4.47633015
C	1.11672218	-1.85821374	4.09848090
C	2.25196788	-0.90303422	4.46169414
C	2.69968150	1.45582648	3.72671688
C	1.34966252	2.01783191	4.16864203
C	-0.11689369	2.93129863	2.39939076
C	-1.06168769	-2.09539421	2.97199322
C	3.88114204	-0.42079596	2.66766136
C	-0.97976877	1.16485004	3.84577511
H	-1.55581344	0.23833011	0.98636430
H	0.14154423	-3.26232949	-2.94411668
H	-0.58094415	-1.56983607	-4.72440789
H	-1.67690774	-1.81284524	-3.35848934
H	1.85616774	2.33454064	-3.42274620
H	0.16212427	2.76037237	-3.29469061
H	0.36214788	0.93630997	-4.95299362
H	1.38539150	0.01807574	-3.83824614
H	-3.89573517	1.03886799	-2.97845802
H	-3.46948591	-0.65824689	-3.06917911
H	-2.14456609	0.47703239	-4.78615282
H	-1.75552679	1.79299242	-3.67112312
H	-1.68531745	-3.18884486	-0.74997194
H	-3.13994575	-5.08676251	-0.10032687
H	-2.20590884	-6.97204091	1.21730583
H	0.18707807	-6.96454691	1.86641568
H	1.65007318	-5.09663584	1.19292186
H	1.74015167	-4.93179930	-2.01428859
H	3.84715139	-5.91281089	-2.81370413
H	6.02258945	-4.96220597	-2.09553811
H	6.07414682	-2.99963554	-0.57870052
H	3.95817259	-1.98048442	0.19962776
H	3.45388523	-0.32137964	-1.52551879
H	5.55321172	-0.75597326	-2.73824767

H	6.99671734	1.13672277	-3.45155202
H	6.32033923	3.46612063	-2.93865491
H	4.23323198	3.91331573	-1.71738202
H	3.81735872	3.83149214	0.96514104
H	3.66379737	6.21590435	1.57315616
H	1.78994152	7.60227683	0.72535263
H	0.05738071	6.58795628	-0.72858331
H	0.18807293	4.18852782	-1.32578973
H	-2.91801566	3.03316655	-1.34153731
H	-3.97927815	5.27544530	-1.34460261
H	-6.17761473	5.60765114	-0.24877289
H	-7.31749576	3.69807490	0.84772944
H	-6.28010837	1.46219188	0.84037309
H	-6.07325785	0.20277682	-1.98131806
H	-7.75649353	-1.57385107	-2.23807659
H	-7.70489017	-3.56701203	-0.76414705
H	-5.94987483	-3.77573007	0.97576925
H	-4.23281633	-2.00248340	1.22474172
H	-1.65038563	-0.62782671	4.81500804
H	1.45320529	-2.57599012	3.35449231
H	3.13283994	-1.48029815	4.74589524
H	1.99253846	-0.29881191	5.32817385
H	3.43218409	1.56609169	4.53317388
H	3.06358429	1.99878171	2.85840653
H	1.06710066	1.62446094	5.14235242
H	1.43587714	3.09873005	4.28990368
H	0.77236285	3.36865983	1.96390124
H	-0.59488910	3.66370895	3.05583976
H	-0.80354080	2.65908521	1.60440429
H	-1.87697784	-1.54970213	2.50366376
H	-1.45653930	-2.69953260	3.79386069
H	-0.60114832	-2.74684824	2.24230984
H	3.79580172	-1.46448611	2.38252892
H	4.71531265	-0.30090077	3.36411535
H	4.05419819	0.17764603	1.77866793
H	-1.33237636	1.86945483	4.60681798
H	-1.75710355	1.08527785	3.08703218
H	0.79595049	-2.41186054	4.98733500
H	-0.08149095	-0.09794768	5.35908923
H	1.36229892	-2.07000295	-3.34106173
Mn	-0.56248104	0.15068885	-1.24833080

Table S6. [(TMTACN)Fe^{III}–(μ-OH)–Mn^{III}poat]$^{2+}$, F (5/2, 2) optimized.

Fe	0.98773108	0.02442754	1.79589007
P	1.11836294	-2.59983366	-0.30722160
P	2.10800510	2.06124362	-0.67502667
P	-3.71112313	0.48010129	-0.16967579
O	-0.56511994	0.23298266	0.65713370
O	1.45663743	-1.70578318	0.95279804
O	2.07457903	1.22270642	0.66512284
O	-2.99469616	0.26042994	1.19394088
N	-0.67277338	0.04118876	-3.30847288
N	0.37967182	-1.69442984	-1.46381124
N	0.86552179	1.59892284	-1.64243098
N	-2.57909528	0.38127419	-1.40540442
N	2.61871385	-0.00135064	3.30366920
N	0.28871425	1.70640035	3.17754169
N	-0.03048754	-1.11923536	3.49678027
C	0.37583506	-2.18470886	-2.87311529
C	-0.70275471	-1.43071741	-3.64566933
C	0.87122435	1.97402919	-3.08752351
C	0.53861949	0.72816363	-3.90022910
C	-3.08764191	0.33880078	-2.80755185
C	-1.94808426	0.73040887	-3.73688209
C	0.05043869	-3.99573510	0.18184876
C	-1.30824028	-3.99505129	-0.17861648
C	-2.13768261	-5.05431476	0.19833009
C	-1.61974921	-6.12022731	0.93904450
C	-0.26850773	-6.12919533	1.30323991
C	0.56522713	-5.07634841	0.92456941
C	2.67014610	-3.37640295	-0.87221399
C	2.63905991	-4.50458327	-1.71470608
C	3.82720344	-5.08332222	-2.16352407
C	5.05925010	-4.55344094	-1.76883239
C	5.10052114	-3.44058568	-0.92455314
C	3.91353024	-2.85288763	-0.47995721
C	3.69845427	1.79874335	-1.52588210
C	4.06430894	0.47663275	-1.84632131
C	5.24200038	0.21887781	-2.54648206
C	6.06593727	1.27657101	-2.94771636
C	5.70521561	2.59157611	-2.64897735
C	4.52738746	2.85591611	-1.94344604
C	2.04405813	3.82798905	-0.23381361
C	3.03393591	4.40094346	0.59005492
C	2.96756683	5.75111914	0.93581979
C	1.91550358	6.54529190	0.46455970
C	0.92864119	5.98500342	-0.34961446

C	0.99015485	4.63219604	-0.69636823
C	-4.52330824	2.10804969	-0.24740268
C	-3.87800934	3.19438584	-0.86423227
C	-4.46830503	4.45970686	-0.86153518
C	-5.70484132	4.65416608	-0.23958400
C	-6.35033072	3.58155915	0.38290484
C	-5.76649206	2.31384278	0.37903348
C	-5.01913218	-0.77234322	-0.37101783
C	-6.02935498	-0.64747637	-1.34301383
C	-6.99730404	-1.64361079	-1.48500140
C	-6.97372973	-2.76761205	-0.65420112
C	-5.98120006	-2.89354019	0.32212346
C	-5.00664901	-1.90327980	0.46406320
C	-0.68506330	-0.18205639	4.48261715
C	1.12808553	-1.86214656	4.10006554
C	2.27148231	-0.91474535	4.45792657
C	2.73376120	1.44067343	3.71966756
C	1.38952473	2.01235756	4.16676285
C	-0.07544951	2.93429391	2.40020636
C	-1.05628625	-2.08397264	2.98115247
C	3.89635148	-0.44590101	2.65558892
C	-0.94727877	1.17582962	3.85117098
H	-1.54419332	0.24762549	0.99998494
H	0.13963580	-3.25076881	-2.95075188
H	-0.58603018	-1.55783007	-4.72535023
H	-1.68346148	-1.80438129	-3.36135561
H	1.84173718	2.34976113	-3.42219706
H	0.14772022	2.77105761	-3.28008745
H	0.34388484	0.95788008	-4.95134678
H	1.37220708	0.03385754	-3.84619939
H	-3.90817430	1.04494317	-2.96827624
H	-3.47959912	-0.65166589	-3.05687863
H	-2.16278851	0.48408478	-4.78026382
H	-1.77111650	1.80144668	-3.66797713
H	-1.70870918	-3.16410548	-0.74259465
H	-3.17943696	-5.04868859	-0.09071358
H	-2.26017927	-6.94322631	1.22411374
H	0.13410026	-6.95808793	1.86847610
H	1.61290370	-5.10371950	1.19258900
H	1.69634080	-4.94984612	-2.00183094
H	3.79156651	-5.95126715	-2.80672564
H	5.97789710	-5.01112120	-2.10800100
H	6.05220385	-3.03833980	-0.60542386
H	3.94826755	-1.99926987	0.17867033
H	3.42722505	-0.34528574	-1.55331898
H	5.51390215	-0.80101863	-2.78046376

H	6.97744698	1.07640214	-3.49324702
H	6.33418589	3.41175115	-2.96517765
H	4.26075118	3.87976118	-1.72865976
H	3.85953697	3.80067590	0.94940623
H	3.73519212	6.18578650	1.56091667
H	1.87018946	7.59273143	0.72758237
H	0.11678761	6.59816575	-0.71501964
H	0.21768716	4.19810934	-1.31500649
H	-2.91854962	3.04301020	-1.34063298
H	-3.97147313	5.28925488	-1.34598633
H	-6.16395793	5.63289549	-0.24195128
H	-7.30623883	3.73072890	0.86498276
H	-6.27697607	1.49104613	0.86000598
H	-6.08160838	0.23163175	-1.97095689
H	-7.77360850	-1.53647489	-2.22962341
H	-7.73141307	-3.53168538	-0.75817441
H	-5.97727679	-3.75087703	0.98135060
H	-4.25144056	-1.98601219	1.23201360
H	-1.62784355	-0.61069342	4.82466891
H	1.45641289	-2.58316973	3.35558315
H	3.14971061	-1.49758925	4.73868924
H	2.01981097	-0.30769548	5.32463053
H	3.47055432	1.54501393	4.52286118
H	3.09813190	1.98079612	2.84974984
H	1.10776591	1.62128513	5.14160784
H	1.48365281	3.09266171	4.28715852
H	0.81677307	3.36868951	1.96789303
H	-0.55280143	3.66792111	3.05568827
H	-0.75982135	2.66400427	1.60275608
H	-1.86929146	-1.53266243	2.51547355
H	-1.45221876	-2.68452582	3.80508290
H	-0.60340958	-2.73921221	2.25010875
H	3.80112269	-1.48867795	2.37020791
H	4.73408168	-0.33306674	3.34877532
H	4.07075191	0.15166894	1.76624355
H	-1.29277002	1.88389589	4.61221433
H	-1.72710097	1.10102951	3.09450513
H	0.80669103	-2.41245293	4.99071594
H	-0.05341847	-0.09151720	5.36326725
H	1.35678497	-2.05214594	-3.33882518
Mn	-0.57638523	0.16000449	-1.24851594

Table S7. [(TMTACN)Fe^{III}–(μ-O)–Mn^{III}Hpoat]$^{2+}$, BS (5/2, –2) optimized.

Fe	-1.39688417	-0.19938984	1.72266398
P	-1.84246298	-1.99476892	-1.11209376
P	-1.15077733	2.62341967	-0.02339784
P	3.96413651	-0.60531294	-0.53398005
O	0.24418740	-0.23085500	0.88911014
O	-2.23353645	-1.30976232	0.24147467
O	-1.82913472	1.66917633	1.03428095
O	2.72653311	0.30522263	-0.31331875
N	1.33342516	0.26376853	-2.93753660
N	-0.32557174	-1.54191439	-1.58819839
N	-0.29374643	1.77051941	-1.14137660
N	3.69835727	-1.65647976	-1.81009922
H	3.73170173	-2.64965561	-1.63593129
N	-0.74513127	0.61163830	3.74357699
N	-1.30158683	-2.10279318	3.01912014
N	-3.39401759	-0.12167296	2.81181493
C	0.45951174	-0.68221505	-3.73856676
H	0.93896868	-0.90332413	-4.69630925
H	-0.47996475	-0.17669374	-3.94663222
C	0.15815721	-1.94350168	-2.93817414
H	-0.57696364	-2.53295459	-3.49311037
H	1.04577511	-2.57112716	-2.83880254
C	0.96098292	1.70998039	-3.23244205
H	0.93311800	1.85879787	-4.31669363
H	1.75574664	2.32854859	-2.81974908
C	-0.35463060	2.10570193	-2.58868523
H	-0.49572869	3.17296017	-2.77163149
H	-1.19656812	1.60027160	-3.07145803
C	2.78511320	0.13094188	-3.35533245
H	3.34127217	0.89120518	-2.81301951
H	2.83809092	0.39765093	-4.41661568
C	3.48392314	-1.22403293	-3.20855333
H	2.93481545	-2.00899943	-3.72569164
H	4.44170912	-1.12078551	-3.72770459
C	-1.95470819	-3.80729291	-0.93490262
C	-0.80484542	-4.61154848	-1.01213516
H	0.15756979	-4.15180491	-1.18738902
C	-0.89851342	-5.99631585	-0.84790180
H	-0.01004795	-6.60879729	-0.91553525
C	-2.13875568	-6.58971151	-0.60029503
H	-2.21142703	-7.66142408	-0.47899326
C	-3.28883868	-5.79728588	-0.51518575
H	-4.24943698	-6.25648892	-0.32778420
C	-3.20042576	-4.41479065	-0.68251128

H	-4.09856617	-3.81389903	-0.63028019
C	-3.07602184	-1.54256423	-2.37686339
C	-3.49006390	-2.43772352	-3.38024530
C	-4.38154341	-2.02045583	-4.37317323
C	-4.86897256	-0.71180970	-4.37447412
C	-4.46212800	0.18521653	-3.38055568
H	-4.83990919	1.19840719	-3.37681064
C	-3.57050228	-0.22517069	-2.38947614
H	-3.25993044	0.46732273	-1.62108072
C	-2.44859371	3.64328216	-0.80263956
C	-3.78507491	3.21121739	-0.80980897
H	-4.05168682	2.28770805	-0.31958159
C	-4.77596946	3.97932747	-1.42586536
H	-5.80371481	3.64331455	-1.41145187
C	-4.44332963	5.18707510	-2.04502925
H	-5.21100424	5.78381584	-2.51722983
C	-3.11732173	5.62951807	-2.04021007
H	-2.85802233	6.56964426	-2.50645750
C	-2.12559842	4.86703519	-1.42052134
H	-1.11040192	5.23911202	-1.39940422
C	-0.06648869	3.81893587	0.82957734
C	-0.62157386	4.86594970	1.59145315
H	-1.69499560	4.98240429	1.65821905
C	0.20748693	5.77447676	2.25068764
H	-0.22685776	6.58130114	2.82448712
C	1.59793765	5.64880060	2.15926823
H	2.23861031	6.35797109	2.66472553
C	2.15564276	4.61218542	1.40664587
H	3.23061920	4.51843854	1.33278463
C	1.33014319	3.70162840	0.74095860
H	1.76241537	2.89681099	0.16356645
C	5.39974851	0.42882792	-0.90708851
C	5.45615886	1.74025357	-0.40104315
H	4.62676107	2.12853358	0.17260695
C	6.57828986	2.53329832	-0.64355957
H	6.62071304	3.53996422	-0.25220921
C	7.64792779	2.02847608	-1.38939535
H	8.51605862	2.64531040	-1.57388773
C	7.59666178	0.72749243	-1.89835614
C	6.47827250	-0.07283801	-1.65949295
H	6.44461196	-1.07651880	-2.06204737
C	4.29470204	-1.67577213	0.88353236
C	5.48691311	-2.41679816	0.98319563
H	6.23836205	-2.36324069	0.20745079
C	5.72122867	-3.21214225	2.10464508
H	6.63890785	-3.77771055	2.18027965

C	4.77763916	-3.26398924	3.13526753
H	4.96730546	-3.87277814	4.00816535
C	3.59741503	-2.52181371	3.04206680
H	2.87867632	-2.55815308	3.84954419
C	3.34851625	-1.72571714	1.92135926
H	2.44080849	-1.14165845	1.83927893
C	0.05111461	-0.49729508	4.36252527
H	0.35666038	-0.22673041	5.37971529
H	0.95411141	-0.60199892	3.76292480
C	-0.73047193	-1.80239397	4.37766671
H	-0.07817362	-2.61878470	4.69469343
H	-1.53652396	-1.76111065	5.10615440
C	-0.47446338	-3.14839175	2.34490087
H	-0.87920499	-3.35429952	1.36365393
H	-0.47087379	-4.07379592	2.92962950
H	0.54341257	-2.78783341	2.23104428
C	-2.73796646	-2.53349505	3.08965743
H	-2.84624399	-3.40159711	3.74999423
H	-3.02161618	-2.83329045	2.08485731
C	-3.64031176	-1.39908882	3.57250595
H	-4.68301513	-1.70061419	3.45689684
H	-3.49859102	-1.21342808	4.63559852
C	-4.50262731	0.12284326	1.83784535
H	-4.27499601	1.01965655	1.27333080
H	-5.45696739	0.24824253	2.35827264
H	-4.56303272	-0.71435431	1.14979549
C	-3.22786448	1.06832248	3.71261007
H	-4.10113256	1.18371473	4.36464473
H	-3.16986791	1.93664619	3.06163992
C	-1.96163302	0.97210501	4.56470250
H	-1.79444342	1.92979126	5.06054253
H	-2.08347743	0.23882245	5.35960613
C	0.14465920	1.80121184	3.56574140
H	0.92641889	1.55958917	2.85216421
H	0.59224872	2.08980022	4.52164444
H	-0.43353306	2.62881984	3.17605454
H	8.42208823	0.33930031	-2.47807411
H	-4.69801715	-2.71789730	-5.13606185
H	-5.56271151	-0.39393812	-5.14015640
H	-3.13779534	-3.45957704	-3.38329458
Mn	0.73693963	0.01296176	-0.81398150

Table S8. [(TMTACN)Fe^{III}–(μ-O)–Mn^{III}Hpoat]$^{2+}$, F (5/2, 2) optimized.

Fe	-1.40884971	-0.18845559	1.72026434
P	-1.86561008	-1.97622441	-1.11281570
P	-1.12057582	2.62309473	-0.02773541
P	3.96867365	-0.63265299	-0.52990402
O	0.25816458	-0.25662240	0.92693467
O	-2.25039785	-1.28911787	0.24271183
O	-1.81371220	1.67642801	1.03039915
O	2.73486342	0.28619976	-0.31635548
N	1.33818842	0.23067918	-2.94399832
N	-0.33919366	-1.54927308	-1.57845458
N	-0.27670925	1.75799040	-1.14300684
N	3.69557804	-1.68602490	-1.80294101
H	3.73191459	-2.67871321	-1.62610849
N	-0.74511617	0.61248796	3.73914405
N	-1.33774914	-2.09415749	3.01414107
N	-3.39895163	-0.08688413	2.81290582
C	0.46324641	-0.72148230	-3.73640849
H	0.94602139	-0.95835653	-4.68868619
H	-0.47241777	-0.21392793	-3.95648193
C	0.15099404	-1.97104955	-2.92086411
H	-0.58182760	-2.56581813	-3.47314504
H	1.03612204	-2.59999332	-2.80743574
C	0.96162974	1.67437929	-3.24485560
H	0.92605118	1.81681025	-4.32975666
H	1.75789408	2.29699204	-2.84107459
C	-0.35105132	2.07233486	-2.59472833
H	-0.50005540	3.13588616	-2.79235472
H	-1.19350025	1.55476026	-3.06347793
C	2.78933334	0.09564730	-3.36103230
H	3.34579348	0.85871410	-2.82271287
H	2.84387892	0.35534085	-4.42399651
C	3.48548808	-1.25976378	-3.20439608
H	2.93534820	-2.04651685	-3.71791192
H	4.44466518	-1.16209426	-3.72199335
C	-2.01170942	-3.78768889	-0.94495533
C	-0.87363972	-4.60986589	-1.00717355
H	0.09813249	-4.16449631	-1.16693518
C	-0.99139049	-5.99333309	-0.84813949
H	-0.11179281	-6.61959045	-0.90451288
C	-2.24435179	-6.56753846	-0.61991255
H	-2.33571768	-7.63824120	-0.50235054
C	-3.38287864	-5.75724585	-0.54926951
H	-4.35314804	-6.20170208	-0.37671731
C	-3.27041330	-4.37595117	-0.71222844

H	-4.15976512	-3.76122780	-0.67207103
C	-3.09058484	-1.49769586	-2.37634764
C	-3.50243963	-2.37656435	-3.39514983
C	-4.38503602	-1.94079388	-4.38792067
C	-4.86611050	-0.62979325	-4.37374911
C	-4.46186893	0.25081213	-3.36429464
H	-4.83483933	1.26565727	-3.34849995
C	-3.57890663	-0.17811937	-2.37323528
H	-3.27009709	0.50120669	-1.59263387
C	-2.40371743	3.66470741	-0.80259280
C	-3.74941088	3.26244913	-0.79818362
H	-4.03277147	2.34773741	-0.30104712
C	-4.72750468	4.04908358	-1.41143250
H	-5.76236326	3.73619561	-1.38783227
C	-4.37257195	5.24560728	-2.03987333
H	-5.13020089	5.85660321	-2.51006318
C	-3.03701468	5.65845967	-2.04697110
H	-2.76023800	6.58988767	-2.52056922
C	-2.05820134	4.87773878	-1.42963616
H	-1.03491280	5.22751606	-1.41730778
C	-0.02383336	3.80361259	0.83012037
C	-0.56850260	4.85588398	1.59235526
H	-1.64067958	4.98402199	1.65782375
C	0.26935401	5.75484508	2.25349209
H	-0.15701802	6.56574629	2.82753248
C	1.65849654	5.61449632	2.16340627
H	2.30613814	6.31622350	2.67038058
C	2.20586375	4.57320615	1.40972349
H	3.27987040	4.46841344	1.33653014
C	1.37152286	3.67217027	0.74204268
H	1.79607870	2.86462768	0.16288572
C	5.40997434	0.39237961	-0.90424909
C	5.47367897	1.70470063	-0.40139611
H	4.64625254	2.09921569	0.17081666
C	6.60049657	2.49055601	-0.64538569
H	6.64854417	3.49798760	-0.25668337
C	7.66754571	1.97759944	-1.38941252
H	8.53931623	2.58893993	-1.57502393
C	7.60899738	0.67570103	-1.89522552
C	6.48582978	-0.11751419	-1.65501447
H	6.44648858	-1.12194164	-2.05523437
C	4.28683439	-1.69910182	0.89264955
C	5.48608077	-2.42568593	1.01240489
H	6.25149089	-2.35982395	0.25140809
C	5.70852111	-3.22226222	2.13534956
H	6.63149260	-3.77676778	2.22680619

C	4.74567053	-3.28960877	3.14706658
H	4.92570625	-3.89967793	4.02113171
C	3.55842568	-2.56148727	3.03364478
H	2.82445492	-2.61050272	3.82662798
C	3.32080288	-1.76363635	1.91166518
H	2.40740422	-1.18970843	1.81530949
C	0.03888030	-0.50716583	4.35485726
H	0.35020158	-0.24085669	5.37136440
H	0.93759993	-0.62299431	3.75159219
C	-0.76000097	-1.80138279	4.37167387
H	-0.11817414	-2.62656861	4.68709504
H	-1.56419053	-1.74940143	5.10150878
C	-0.52461649	-3.14946043	2.33751485
H	-0.93802824	-3.35400309	1.35978036
H	-0.52674687	-4.07282219	2.92537135
H	0.49486353	-2.79745596	2.21475321
C	-2.77909252	-2.50724326	3.08797970
H	-2.89645090	-3.37421313	3.74811203
H	-3.06928905	-2.80292122	2.08377648
C	-3.66378280	-1.36086282	3.57403046
H	-4.71127069	-1.64578577	3.46035616
H	-3.51646235	-1.17753557	4.63670606
C	-4.50432737	0.17087915	1.83783408
H	-4.26528998	1.06408407	1.27244589
H	-5.45664486	0.30888796	2.35849430
H	-4.57549401	-0.66643766	1.15094656
C	-3.21970382	1.10125525	3.71417340
H	-4.09160953	1.22538313	4.36617806
H	-3.15256890	1.96927314	3.06366068
C	-1.95369702	0.98930011	4.56424797
H	-1.77251058	1.94487169	5.05909475
H	-2.08311239	0.25779114	5.35956047
C	0.16109927	1.78965780	3.55850650
H	0.93340328	1.53656084	2.83902837
H	0.61798921	2.06869315	4.51278819
H	-0.40730296	2.62680114	3.17481284
H	8.43242147	0.28131840	-2.47359899
H	-4.70012106	-2.62579693	-5.16257437
H	-5.55323479	-0.29769119	-5.13936067
H	-3.15597034	-3.40029730	-3.40985335
Mn	0.75779766	-0.00806000	-0.81580008

Table S9. [(TMTACN)Fe^{III}–(μ-O)–Mn^{IV}poat]$^{2+}$, BS (5/2, –3/2) optimized.

Fe	0.75995185	0.19194049	1.83288696
P	1.43853478	-2.48798618	-0.06936197
P	1.85032071	2.11862607	-0.73923198
P	-3.56314956	-0.11037912	-0.12335759
O	-0.45348428	0.15865406	0.45458507
O	1.59973960	-1.53081097	1.15447938
O	1.92230040	1.44478682	0.66878683
O	-3.05253055	-0.69624382	1.18368000
N	-0.55909545	-0.39532664	-3.34780349
N	0.60403345	-1.74670237	-1.32411914
N	0.75181734	1.34497198	-1.73916883
N	-2.39771064	-0.12906360	-1.42422469
N	2.31198053	0.55291482	3.43419778
N	-0.25178664	1.82270775	2.98832664
N	-0.12383985	-0.99006801	3.53922601
C	0.70991911	-2.39265645	-2.66801718
C	-0.41722907	-1.87718843	-3.55091199
C	0.77461722	1.66590307	-3.19873223
C	0.57754266	0.37174687	-3.97226972
C	-2.95793229	-0.30168834	-2.80147528
C	-1.89607120	0.11246896	-3.80812164
C	0.54263651	-4.01419277	0.35700695
C	-0.81681545	-4.15791144	0.02742547
C	-1.50691809	-5.31719840	0.38628264
C	-0.85145029	-6.33816748	1.08049900
C	0.49891422	-6.20056800	1.41767214
C	1.19682426	-5.04789424	1.05530711
C	3.10715817	-3.03129046	-0.57063722
C	3.29876851	-4.12264077	-1.44018559
C	4.58720511	-4.53012417	-1.78857470
C	5.70008947	-3.86570692	-1.26436974
C	5.52094259	-2.79185098	-0.38835983
C	4.23293033	-2.37545784	-0.04307293
C	3.48359391	2.07925712	-1.54526547
C	4.14961318	0.84208611	-1.63243554
C	5.38014080	0.74625415	-2.28062637
C	5.96044510	1.88164074	-2.85694193
C	5.30370533	3.11152430	-2.78489469
C	4.06995068	3.21400246	-2.13549760
C	1.41384352	3.87612221	-0.54838235
C	2.25766027	4.72019579	0.20219416
C	1.92992525	6.06431867	0.38111458
C	0.76040835	6.58300498	-0.18620896
C	-0.08363685	5.75227830	-0.92643727

C	0.23875155	4.40416796	-1.10554003
C	-4.10065520	1.62690950	0.03719923
C	-3.72568810	2.64332788	-0.85561921
C	-4.20589574	3.94545680	-0.68675318
C	-5.06048550	4.24474653	0.37722512
C	-5.43311319	3.24038658	1.27682880
C	-4.95898614	1.93872603	1.10949713
C	-5.01112127	-1.05005880	-0.72107420
C	-6.07800410	-0.45502591	-1.41728831
C	-7.16577392	-1.22759131	-1.83126398
C	-7.20051954	-2.59650685	-1.55259352
C	-6.15063023	-3.19290537	-0.84725823
C	-5.06214278	-2.42576718	-0.42904783
C	-1.01350083	-0.10516783	4.37837567
C	1.08657768	-1.46610578	4.28946962
C	2.02202660	-0.31100843	4.63928534
C	2.14844575	2.01827382	3.72495638
C	0.69334223	2.38204122	4.02448296
C	-0.73937292	2.90959712	2.08236239
C	-0.92407617	-2.15843902	3.04127601
C	3.69259112	0.28244164	2.92314030
C	-1.44087334	1.12328953	3.59237617
H	0.60471626	-3.47862286	-2.59326392
H	-0.23611088	-2.09830695	-4.60614119
H	-1.35767618	-2.34059478	-3.26185308
H	1.72321887	2.11389004	-3.50178982
H	-0.00777367	2.38938371	-3.44368336
H	0.37001528	0.54960323	-5.03055770
H	1.47562203	-0.23415618	-3.89424132
H	-3.85146868	0.30596496	-2.95548353
H	-3.27351411	-1.33744443	-2.95049045
H	-2.11673169	-0.25791835	-4.81284320
H	-1.83277044	1.19816696	-3.85571386
H	-1.32827093	-3.35784149	-0.48860446
H	-2.55035722	-5.42506181	0.12481168
H	-1.38688473	-7.23639982	1.35433755
H	1.00722753	-6.99084655	1.95186636
H	2.24456792	-4.95919202	1.30741910
H	2.45395067	-4.67647198	-1.82449129
H	4.72193925	-5.37205011	-2.45280904
H	6.69682688	-4.19245232	-1.52539914
H	6.37949431	-2.28986990	0.03596872
H	4.09543701	-1.55937613	0.64858700
H	3.70718715	-0.04043752	-1.19511745
H	5.88328476	-0.20905846	-2.33611394
H	6.91477557	1.80681637	-3.35902003

H	5.74686953	3.99022822	-3.23178551
H	3.57513608	4.17280910	-2.09272479
H	3.17004534	4.33359442	0.63659647
H	2.58556736	6.70661190	0.95230261
H	0.51288217	7.62676805	-0.05347698
H	-0.98772416	6.15233900	-1.36365063
H	-0.42777007	3.76169705	-1.66191496
H	-3.05784403	2.41927570	-1.67550903
H	-3.92394796	4.71977061	-1.38734367
H	-5.43868580	5.24981219	0.50192336
H	-6.09751973	3.46863064	2.09854297
H	-5.25543195	1.16562504	1.80516850
H	-6.07822095	0.60857397	-1.61414561
H	-7.98691037	-0.76070046	-2.35701795
H	-8.04632359	-3.19083223	-1.86876642
H	-6.18959260	-4.24728892	-0.61066005
H	-4.27004001	-2.87688323	0.15271202
H	-1.89983016	-0.66336171	4.68105452
H	1.59325602	-2.17476557	3.63843773
H	2.95620664	-0.71022280	5.03760182
H	1.59742196	0.30796741	5.42671592
H	2.78540626	2.31502970	4.56555055
H	2.48907407	2.54497004	2.83722316
H	0.39691459	2.02313454	5.00694756
H	0.59834049	3.46862328	4.05638630
H	0.10730356	3.44551559	1.67055603
H	-1.37192252	3.60854231	2.63666832
H	-1.31172155	2.46892279	1.27287510
H	-1.74006785	-1.79372199	2.42230702
H	-1.32056516	-2.72461339	3.88948174
H	-0.28418380	-2.80487803	2.45618119
H	3.79241576	-0.78159826	2.72969199
H	4.44250977	0.58633408	3.65936373
H	3.84049232	0.82790542	1.99705591
H	-1.99613594	1.81480790	4.23509119
H	-2.08641206	0.81912429	2.76868240
H	0.78776698	-1.99419979	5.20128999
H	-0.49145489	0.16525370	5.29365901
H	1.68666328	-2.20031169	-3.11839919
Mn	-0.48340261	-0.10960136	-1.26319387

Table S10. [(TMTACN)Fe^{III}–(μ-O)–Mn^{IV}poat]$^{2+}$, F (5/2, 3/2) optimized.

Fe	0.78339838	0.22562561	1.81058176
P	1.31962693	-2.53569059	-0.00823859
P	1.92529510	2.02795034	-0.81683671
P	-3.57698600	-0.12694953	-0.16019467
O	-0.50235490	0.22037876	0.45553349
O	1.53207520	-1.53615756	1.17633501
O	1.96490048	1.39676439	0.61396962
O	-3.12251593	-0.93295585	1.04580169
N	-0.57148358	-0.46574213	-3.36314613
N	0.51354381	-1.80144929	-1.28358334
N	0.79653483	1.26953081	-1.79041819
N	-2.42494344	-0.10919463	-1.47139377
N	2.34911274	0.58200900	3.39815151
N	-0.16134736	1.92804545	2.91869414
N	-0.13615648	-0.86869908	3.55938859
C	0.59645532	-2.49683873	-2.60542817
C	-0.49551275	-1.95766292	-3.51785662
C	0.84275101	1.54310729	-3.26062120
C	0.60174640	0.23386526	-3.99657420
C	-2.97295814	-0.27409925	-2.85248400
C	-1.88189316	0.08593221	-3.84904617
C	0.36580232	-4.00462801	0.48093743
C	-1.01481948	-4.06913046	0.21909146
C	-1.75411121	-5.17942620	0.63000839
C	-1.12748314	-6.23037127	1.30583311
C	0.24380604	-6.17143478	1.57487252
C	0.99084966	-5.06718968	1.16313588
C	2.96340958	-3.16359760	-0.49433200
C	3.10712951	-4.28242072	-1.33805981
C	4.37627614	-4.75468273	-1.67514789
C	5.51748807	-4.12883360	-1.16456214
C	5.38570219	-3.02781434	-0.31437699
C	4.11729207	-2.54626792	0.01859188
C	3.56093708	1.89187167	-1.60824382
C	4.17639568	0.62637670	-1.64389810
C	5.40604022	0.45596404	-2.27836511
C	6.03576379	1.54401283	-2.89273195
C	5.42907292	2.80123425	-2.87278876
C	4.19669459	2.97847648	-2.23699202
C	1.57108141	3.80954864	-0.68212044
C	2.45316687	4.63885541	0.04049189
C	2.19036330	6.00282161	0.16980839
C	1.04891799	6.55666767	-0.42094004
C	0.16764452	5.74148277	-1.13455149

C	0.42437453	4.37353812	-1.26288002
C	-3.93103563	1.62470246	0.21277597
C	-3.53105957	2.68721438	-0.61266059
C	-3.89620338	4.00054234	-0.30134502
C	-4.66072091	4.26425760	0.83808757
C	-5.05860487	3.21216754	1.66996900
C	-4.70015651	1.89932383	1.35970643
C	-5.12958441	-0.81571318	-0.83422719
C	-6.09210461	-0.03456234	-1.49881505
C	-7.26788740	-0.62053850	-1.97274791
C	-7.49555168	-1.98697487	-1.78579879
C	-6.55075798	-2.76725672	-1.11270959
C	-5.37463392	-2.18650253	-0.63467073
C	-0.99139394	0.07437442	4.36923631
C	1.05917093	-1.36188740	4.32372867
C	2.03673297	-0.23149248	4.63268508
C	2.24388920	2.06094242	3.64360130
C	0.80494670	2.48536824	3.93670279
C	-0.60862438	3.00411739	1.97858708
C	-0.97677624	-2.02891400	3.10814318
C	3.71533272	0.24318362	2.88709917
C	-1.37306050	1.29388796	3.54681950
H	0.43632614	-3.57319438	-2.49588229
H	-0.31308985	-2.22151804	-4.56304796
H	-1.45907230	-2.36836861	-3.22575087
H	1.80918588	1.94570555	-3.57102567
H	0.08964751	2.28711054	-3.53408977
H	0.41344520	0.38827362	-5.06223224
H	1.47353890	-0.40535112	-3.88991429
H	-3.83793020	0.36926858	-3.02161368
H	-3.33181653	-1.29639094	-2.99855188
H	-2.10290809	-0.28913780	-4.85202530
H	-1.77686012	1.16781639	-3.90915865
H	-1.50520171	-3.24388185	-0.27615575
H	-2.81492555	-5.22337402	0.42706511
H	-1.70205418	-7.09055576	1.61974003
H	0.72946340	-6.98468407	2.09551770
H	2.05257592	-5.03860502	1.36511139
H	2.23890409	-4.80688753	-1.71097892
H	4.47356889	-5.61660456	-2.31999983
H	6.49879225	-4.50569409	-1.41632570
H	6.26545318	-2.55472906	0.09964493
H	4.01670603	-1.70728789	0.68882458
H	3.69559970	-0.22038175	-1.17769365
H	5.86971453	-0.52055401	-2.29425594
H	6.98928817	1.41151282	-3.38433183

H	5.91018358	3.64342187	-3.34974491
H	3.74143298	3.95760328	-2.23445726
H	3.34633487	4.22560700	0.49010906
H	2.87535016	6.63302070	0.71963358
H	0.85255302	7.61549658	-0.32789768
H	-0.71328021	6.16931559	-1.59239331
H	-0.26993917	3.74516315	-1.80099908
H	-2.93348236	2.48843549	-1.49152718
H	-3.59559561	4.81235259	-0.94973158
H	-4.95296752	5.27861653	1.07166757
H	-5.65816413	3.41247641	2.54714034
H	-5.02261802	1.08843365	1.99865581
H	-5.94290006	1.02961849	-1.62419123
H	-8.00771678	-0.01177414	-2.47356156
H	-8.40926383	-2.43677154	-2.14830434
H	-6.73722660	-3.81949193	-0.94816816
H	-4.66155763	-2.77735463	-0.07684185
H	-1.89698569	-0.44113103	4.68985595
H	1.53715374	-2.11053427	3.69618210
H	2.95888955	-0.65111683	5.03777458
H	1.64078535	0.42760016	5.40233820
H	2.89627660	2.35841933	4.47178491
H	2.59908531	2.54695630	2.73854025
H	0.49956783	2.16611082	4.93020154
H	0.74685799	3.57485605	3.93588693
H	0.25807864	3.49880699	1.55701177
H	-1.21987659	3.73874262	2.50989346
H	-1.18985334	2.55771722	1.17956318
H	-1.77370294	-1.67419335	2.45877708
H	-1.40062202	-2.53732704	3.97926555
H	-0.35489985	-2.72623781	2.56410083
H	3.77542442	-0.82987155	2.73040816
H	4.48079439	0.54482562	3.60786631
H	3.87632071	0.75082440	1.94192850
H	-1.90040012	2.02477022	4.16875422
H	-2.03176359	0.99728846	2.73219570
H	0.74356210	-1.84846096	5.25265250
H	-0.45930952	0.35572850	5.27534143
H	1.58515398	-2.36751230	-3.05232171
Mn	-0.51147565	-0.12232629	-1.28571707

Table S11. Selected structure parameters for the BS/F state and *J* values of [(TMTACN)Fe^{III}–(*μ*-O{H})–$Mn^{III/IV}${H}poat]$^{+/2+}$ complexes. Distances in Å, angle in degrees, and *J* in cm^{-1} ($J\mathbf{S}_1.\mathbf{S}_2$ convention).[a]

	$Fe^{III}(O)Mn^{III}$	$Fe^{III}(OH)Mn^{III}$	$Fe^{III}(O)Mn^{III}$Hpoat	$Fe^{III}(O)Mn^{IV}$
Mn–O_{bridge}	1.793/1.842	1.891/1.907	1.790/1.830	1.739/1.775
Mn–N (O)[b]	2.018/2.019	2.026/2.021	2.072/2.060	1.921/1.923
Mn–N’	2.127/2.134	2.094/2.097	2.064/2.073	1.966/1.967
Mn–N”	2.092/2.100	2.072/2.075	2.036/2.040	1.967/1.976
Mn–N”’	2.093/2.092	2.066/2.066	2.220/2.219	2.105/2.106
Fe–O_{bridge}	1.808/1.818	1.931/1.937	1.841/1.847	1.837/1.868
Fe–O_{bridge}–Mn	133/130	126/126	132/131	139/136
J	170	40	125	235

[a] N, N’ and N” are equatorial atoms and N”’ is the axial atom of the poat ligand coordinating to Mn. Atom N belongs to the arm of [poat]$^{3-}$ that is not coordinated to Fe. [b] In [$Fe^{III}(O)Mn^{III}$Hpoat]$^{2+}$.

References

(1) L. F. Armarego, C. L. L. Chai, Eds. *Purification of Laboratory Chemicals*, Elsevier, Oxford, UK, 5th edn., 2003.

(2) V. F. Oswald, J. L. Lee, S. Biswas, A. C. Weitz, K. Mittra, R. Fan, J. Li, J. Zhao, M. Y. Hu, E. E. Alp, E. L. Bominaar, Y. Guo, M. T. Green, M. P. Hendrich and A. S. Borovik. Effects of Noncovalent Interactions on High-Spin Fe(IV)–Oxido Complexes, *J. Am. Chem. Soc.* 2020, **142**, 11804–11817.

(3) D. W. Blakesley, S. C. Payne and K. S. Hagen. Spin-State Variation in Solid State and Solution of Mononuclear Iron(II) 1,4,7-Trimethyl-1,4,7-triazacyclononane Complexes, *Inorg. Chem.* 2000, **39**, 1979–1989.

(4) N. G. Connelly and W. E. Geiger. Chemical Redox Agents for Organometallic Chemistry, *Chem. Rev.* 1996, **96**, 877–910.

(5) V. V. Zhdankin, D. N. Litvinov, A. Y. Koposov, T. Luu, M. J. Ferguson, R. McDonald and R. R. Tykwinski. Preparation and structure of 2-iodoxybenzoate esters: soluble and stable periodinane oxidizing reagents, *Chem. Commun.* 2004, 106–107.

(6) V. V. Zhdankin, A. Y. Koposov, D. N. Litvinov, M. J. Ferguson, R. McDonald, T. Luu and R. R. Tykwinski. Esters of 2-Iodozybenzoic Acid: Hypervalent Iodine Oxidizing Regents with a Pseudobenziodoxole Structure, *J. Org. Chem.* 2005, **70**, 6484–6491.

(7) J. J. Curley, R. G. Bergman and T. D. Tilley. Preparation and physical properties of early-late heterobimetallic compounds featuring Ir-M bonds (M = Ti, Zr, Hf), *Dalton Trans.* 2012, **41**, 192–200.

(8) J. R. Hagadorn, L. Que and W. B. Tolman. N-Donor Effects on Carboxylate Binding in Mononuclear Fe(II) Complexes of a Sterically Hindered Benzoate Ligand. *Inorg. Chem.* 2000, **39**, 6086–6090.

(9) D. T. Petasis and M. P. Hendrich. Quantitative Interpretation of Multifrequency Multimode EPR Spectra of Metal Containing Proteins, Enzymes, and Biomimetic Complexes. *Methods Enzymol.* **2015**, *563*, 171-208.

(10) APEX2 Version 2014.11-0, Bruker AXS, Inc.; Madison, WI 2014.

(11) SAINT Version 8.34a, Bruker AXS, Inc.; Madison, WI 2013.

(12) Sheldrick, G. M. SADABS, Version 2014/5, Bruker AXS, Inc.; Madison, WI 2014.

(13) Sheldrick, G. M. SHELXTL, Version 2014/7, Bruker AXS, Inc.; Madison, WI 2014.

(14) International Tables for Crystallography 1992, Vol. C., Dordrecht: Kluwer Academic Publishers.

(15) Spek, A. L. SQUEEZE, *Acta Cryst.* **2015**, *C71*, 9–19.

(16) Spek, A. L. PLATON, *Acta. Cryst.* **2009**, *D65*, 148–155.
